# RNA G-quadruplex forming regions from SARS-2, SARS-1 and MERS coronoviruses

**DOI:** 10.3389/fchem.2022.1014663

**Published:** 2022-11-21

**Authors:** Amani Kabbara, Brune Vialet, Julien Marquevielle, Pierre Bonnafous, Cameron D. Mackereth, Samir Amrane

**Affiliations:** Université de Bordeaux, INSERM U1212, CNRS UMR 5320, ARNA Laboratory, IECB, Bordeaux, France

**Keywords:** coronoviruses, COVID-19, MERS-CoV, SARS-CoV-1, SARS-CoV-2, RNA G-quadruplex structures

## Abstract

COVID-19 (Corona Virus Disease 2019), SARS (Severe Acute Respiratory Syndrome) and MERS (Middle East Respiratory Syndrome) are infectious diseases each caused by coronavirus outbreaks. Small molecules and other therapeutics are rapidly being developed to treat these diseases, but the threat of new variants and outbreaks argue for the identification of additional viral targets. Here we identify regions in each of the three coronavirus genomes that are able to form G-quadruplex (G4) structures. G4s are structures formed by DNA or RNA with a core of two or more stacked planes of guanosine tetrads. In recent years, numerous DNA and RNA G4s have emerged as promising pharmacological targets for the treatment of cancer and viral infection. We use a combination of bioinformatics and biophysical approaches to identify conserved RNA G4 regions from the ORF1A and S sequences of SARS-CoV, SARS-CoV-2 and MERS-CoV. Although a general depletion of G4-forming regions is observed in coronaviridae, the preservation of these selected G4 sequences support a significance in viral replication. Targeting these RNA structures may represent a new antiviral strategy against these viruses distinct from current approaches that target viral proteins.

## Introduction

### SARS, MERS and SARS-2 coronaviruses

RNA viruses represent the majority of the viral pathogens that are able to cause severe diseases in humans. The influenza, dengue, chikungunya and coronaviruses are examples of RNA viruses that are currently threatening different populations all around the world. The SARS coronavirus (SARS-CoV) is responsible of the severe acute respiratory syndrome (SARS). It appeared in 2002 in guangding China (Peiris et al., 2003), (Ksiazek et al., 2003) and 8437 cases were reported in 29 countries. The Middle East Respiratory Syndrome (MERS), caused by the MERS coronavirus (MERS-CoV) was identified in Saudi Arabia in 2012 (Zaki et al., 2012), (de Groot et al., 2013) and 2533 cases were reported in 27 different countries with 35% mortality rate. First appearing in Wuhan, China, in December 2019 (Zhu N et al., 2020), (Li et al., 2020) the COrona VIrus Disease 2019 (COVID-19) is caused by infection of the beta-coronavirus SARS-CoV-2.

**FIGURE 1:**
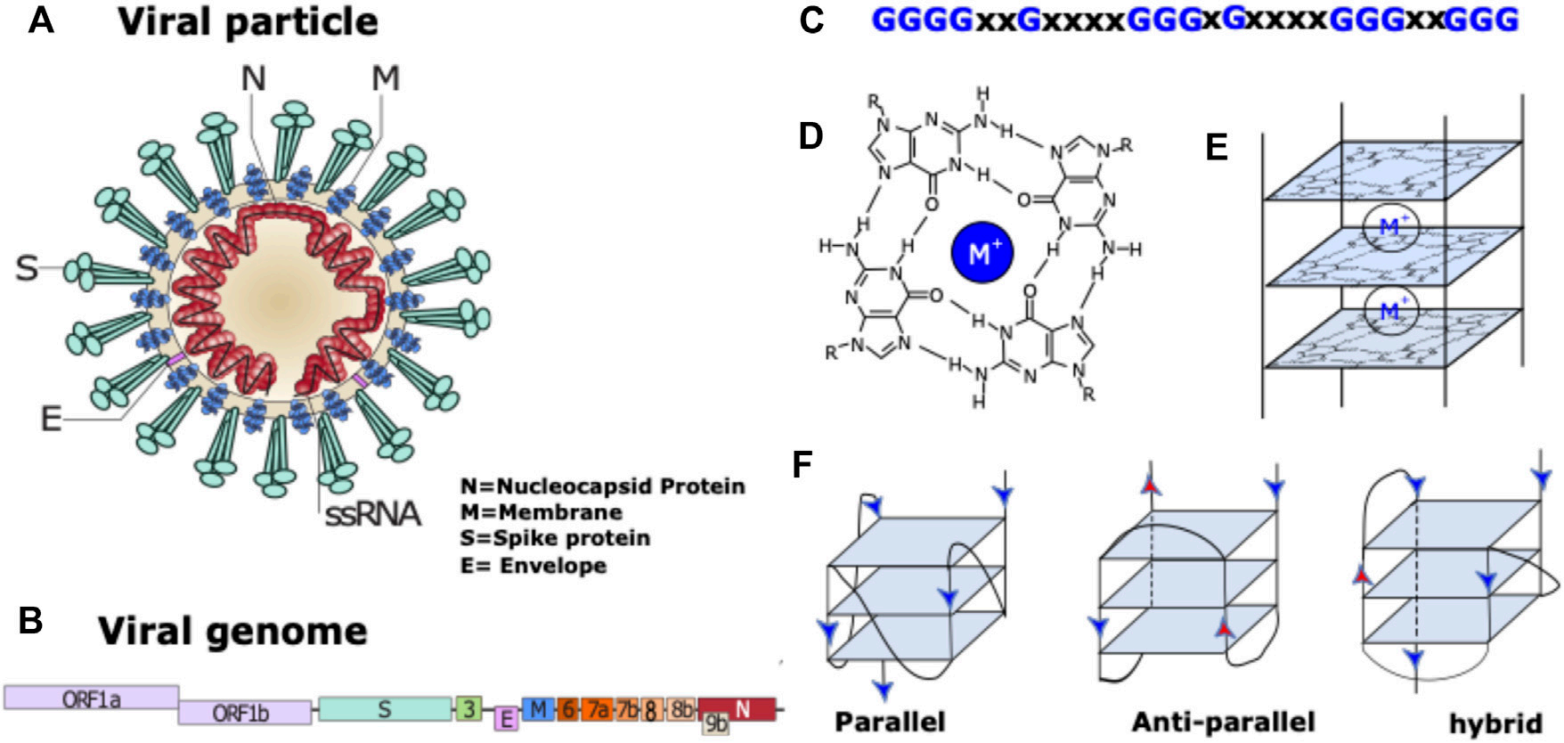
Coronavirus particle and G-quadruplex nucleic acid structures. **(A)** coronavirus particle with its 4 structural proteins. **(B)** Schematic representation of coronavirus genome. **(C)** Example of a G-quadruplex motif: Repeats of two to four guanines are separated by inter-block sequences composed of 1–15 nt (X may be replaced by A, T, G or C). The blocks composed of more than 2 Gs can be interrupted by one or two non-G residues. **(D)** G-quartet structure stabilized by a monovalent cation (M^+^ = Na^+^ or K^+^). **(E)** G-quadruplex structure with 3 quartets. **(F)** Schematic representation of various topologies of intramolecular G-quadruplex structures composed of three tetrads with three different loop types: propeller, diagonal, and lateral.

Coronaviruses are spherical viruses of 0.1 μm diameter, which contain an encapsidated RNA genome of positive polarity (Figures 1A,B). The viral genome (around 30 Kb) encodes three types of viral proteins: structural, non-structural and accessory. In the case of the structural proteins, the RNA is coated by the nucleocapsid protein (N). The membrane (M) protein is responsible for binding both to the nucleocapsid and envelope (E) viral proteins to induce the necessary curvature for viral assembly (Schoeman and Fielding, 2020). The S protein is responsible of the specific binding to the angiotensin-converting enzyme 2 (ACE2) cellular receptor (Letko et al., 2020), (Walls et al., 2020) abundantly present in lung epithelia (Ziegler et al., 2020). The non-structural proteins also called viral replication and transcription complex (RTC) are responsible of the replication of the viral RNA and are encoded by two open-reading frames (ORF1a and ORF1b). The accessory proteins (3, 4, 5, 6, 7, 8 and 9) are not all conserved in the three viruses and are not essential for the viral cycle but play a role in virus host interaction enabling virus adaptation to host (Liu et al., 2014).

### Current pharmacological treatments against COVID-19

Symptoms of the COVID-19 virus vary from asymptomatic to very severe and serious symptoms including fever, cough, chest tightness, headaches and other critical symptoms that might lead to death from the disease (Cui et al., 2019) (Ganesh et al., 2021) (WHO reported that 14.9 million excess deaths are associated with the COVID-19). New variants of this virus are emerging through the countries making this virus a difficult threat even after more than 2 years of the date of its emergence (Zhou and Wang, 2021). Although many vaccines have been developed in record times no pharmaceutical therapeutic medications have been proven effective to treat the COVID-19 virus (Zhu F. C et al., 2020).

Several strategies are currently used to treat COVID-19 and other coronavirus diseases. These treatments are mainly based on repurposed drugs that block the viral cycle at different stages. For instance, Imatinib (Sisk et al., 2018), an Abelson kinase inhibitor is used to inhibit viral endocytosis. The viral proteases papain-like protease (PL^pro^) and chymotrypsin-like protease (3CL^pro^) (Thiel et al., 2003), responsible for the proteolytic processing of the viral proteins, have been targeted using a combination of Lopinavir and Ritonavir initially used for treating HIV, but have shown poor results against COVID-19 (Dong et al., 2020) Molnupiravir (Kabinger et al., 2021) and Remdesivir (de Wit et al., 2020), (Wang et al., 2020) are synthetic nucleoside analogues that inhibit viral RNA polymerases are also used against COVID-19. Among the very recent treatments, Nirmatrelvir is used as a specific 3C-like protease inhibitor (Owen et al., 2021). Finally, several Anti-SARS-CoV-2 monoclonal antibodies that target the spike protein have been developed. They have shown potent effects against SARS-CoV-2 infections, but these effects are strongly dependent on the circulating variant (Taylor et al., 2021). Despite these new approaches, COVID-19 is still progressing. Finding new strategies against this virus is still high priority. In this context, targeting RNA G4 structures may become an interesting avenue.

### Structure and biological functions of G-quadruplex nucleic acids

G-quadruplex nucleic acid structures (or G4s) are non-canonical nucleic acid secondary structures that can be formed by guanine rich DNA or RNA sequences (Jana et al., 2021) in which guanines are organized in several guanines arrays (Figure 1C). To be able to form a G-quadruplex, 2 or more square planar arrangements of 4 guanines (G-quartets) connected through a Hoogsteen-type hydrogen bond needs to be stacked on top of each other (Figures 1D,E). The abundant presence of K^+^ or Na^+^ cations in the cellular environment stabilizes these structures due to specific electrostatic interactions with the oxygen of the carbonyl groups of the four guanines. Depending on the direction of each guanine column, G4s can adopt different conformations: The parallel conformation when all the G-columns point in the same direction, the anti-parallel one, when 2 adjacent G-columns point in opposite direction and the hybrid one when 3 G-columns point in the same direction and one in opposite direction (Figure 1F).

G4s have been gaining more interest in recent years. Many studies proved their impact on various biological processes such as gene expression mechanisms (Lago et al., 2021). The presence of G4s in the human genome and the role they play in disease modulation have been inspected and reviewed significantly in recent years mainly focused on their biological impact in telomeres (Bryan, 2020) and oncogene promoters (Brázda et al., 2021). In addition, it has been shown *in vitro* as well as in cultured cells that G4s play an important role in translational regulation when present in RNA coding regions that stimulate ribosomal frame-shifts (Cammas and Millevoi, 2016).

G-quadruplexes have already been identified in other viral genomes (Amrane et al., 2014) and many studies have emphasized their role as pharmacological targets (Abiri et al., 2021), (Métifiot et al., 2014). Over a thousand G4 ligands have been portrayed in the G4 ligand database (Li et al., 2013) where at least one was tested in clinical trials for anticancer strategies (Onel et al., 2014) These small ligands that are able to bind the G4s present in viral genomes can be a potential new class of therapeutic agent to combat viral infections. Several G4 ligand families such as bisquinoliniums, naphthalene diimides and acridiniums had an inhibiting effect over DNA viruses such as herpesvirus HSV (Callegaro et al., 2017), EBV(Murat et al., 2014) and Hepatitis B virus (Biswas et al., 2017) as well as the RNA viruses HIV-1 and Hepatitis C virus (Jaubert et al., 2018). In contrast to classical antiviral approaches aimed at targeting viral proteins, the advantage of this strategy is that the viral RNA, the actual source of the disease, is directly targeted.

In this study, we identified evolutionary conserved G4 forming sequences in the genomes and negative RNA strands of SARS-CoV-2, SARS-CoV and MERS-CoV by using a combination of bioinformatics, Circular Dichroism, UV-melting, and NMR. Targeting these viral RNA structures may represent a promising new antiviral strategy.

## Results

### *In silico* detection of putative G4 forming sequences

One important feature of RNA viruses is that they evolve extremely rapidly with up to 30% divergence at the sequence level between closely related subspecies (Firth, 2014). Sequence conservation relates to important residues in the encoded proteins, but also to conserved RNA secondary structures (Kiening et al., 2019) that have key functions during the virus life cycle. In HIV-1, such RNA structures include the TAR, DIS or RRE structural elements. In this study, we set out to identify possible G4-containing structural elements in the three coronavirus genomes based on bioinformatic prediction, sequence conservation and biophysical characterization.

**FIGURE 2:**
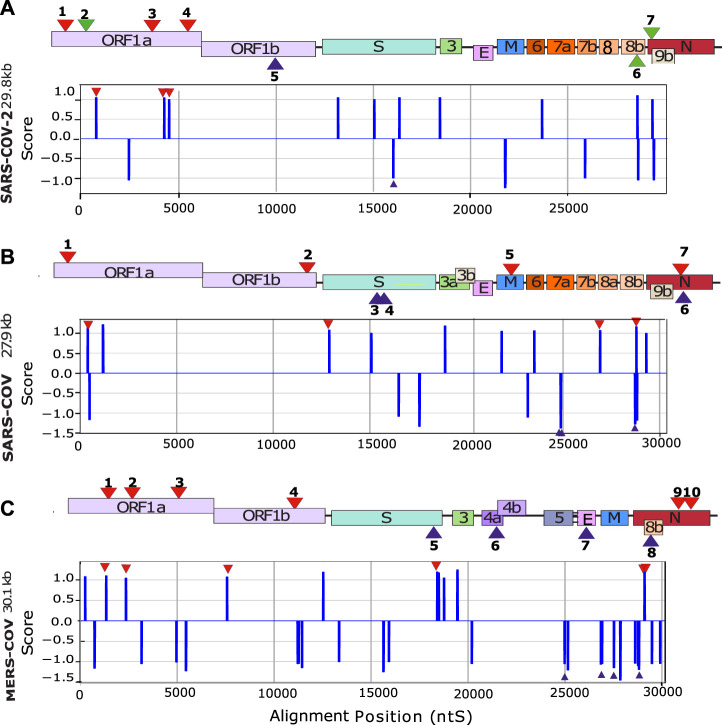
*In silico* detection of putative G4-forming sequences in coronaviruses. Scheme of SARS-CoV-2 **(A)** SARS-CoV **(B)** and MERS-CoV **(C)** genomes. In order to visualize all the genes, their sizes are not scaled to their actual lengths. The positions of the putative G4FSs are indicated by red triangles for sequences located on the (+) RNA and blue triangles indicate those located on the (−) RNA strand. Green triangles indicate putative G4FS detected by other softwares (Table 1). Putative G4FSs are detected on sequence alignments (SARS-2 = 2500 sequences, SARS = 308 sequences and MERS = 459 sequences) using G4Hunter. G4Hunter is a scoring algorithm that assigns each nucleotide with a score over a chosen window (20 nucleotides in this study). A positive score is given for the Gs and a negative for the Cs, whereas the other bases have a score of 0. The G4Hunter score is then calculated for each 20 nucleotides window. The average of the scores for each window of the alignment is then assigned to the first nucleotide of the sequence. Regions with scores greater than or equal to 1 (in absolute value) can form a G4, whereas those with scores below 1 (in absolute value) are unlikely to form a G4.

The first step was to conduct an *in silico* screening of coronavirus genomes to detect the presence of putative G4 forming sequences (putative G4FS). We used the G4-Hunter scoring algorithm to search for putative G4FS over alignments of 2500 SARS-CoV-2, 308 SARS-CoV and 459 MERS-CoV viral genomes retrieved from the NCBI database. To avoid missing any possible G4 forming sequences (G4FS), we used high sensitivity settings for the G4-hunter algorithm with a threshold score of 1 and a sliding window of 20 nucleotides. As a result, we detected 16 putative G4FS in SARS-CoV-2, 21 in SARS-CoV and 40 in MERS-CoV (Figure 2 and Supplementary Tables S1–S3). All identified sequences are highly conserved in the alignments as indicated by the logo representation of each putative G4FS (Supplementary Figures S1–S3). Due to emphasizing high sensitivity there is a corresponding drop in accuracy of the detection with an increased rate of false positives. Therefore, we manually inspected the 77 putative G4FS from the three coronaviruses (Supplementary Tables S1–S3) and discarded the sequences that presented single runs of 4–5 guanines. Indeed, despite a G4H-score>1, these sequences are unlikely to form mono-molecular G4 structures but will tend to form tetra-molecular G4s. We finally selected 24 putative G4FS from the 3 viruses (Figure 2; Table 1) to undergo *in vitro* biophysical validation. In order to evaluate the actual propensity of these sequences to form G4s, we synthesized the putative G4FS and analysed them by using the complementary techniques of Circular Dichroism (CD), Thermal Differential Spectra (TDS), UV-melting and ^1^H-1D NMR. The results are summarized in Table 1 and described in detail in the following sections and in Supplementary Figures S4–S27.

**TABLE 1:** Biophysical analysis of the putative G4FS.

	Name	Sequence (5′-3′)^a^	Length (nt)	RNA strand	Position (nt)	CD^b^	TDS^c^	T_m_ (°C)^d^	NMR^e^	G4?^f^
SARS-CoV-2 (NC_045512.2)										
1	S2-1	GCG​UGA​GCU​UAA​CGG​AGG​GGC	21	(+)	786	Folded	(+)	40°C (295 nm)	YES	YES
2	S2-2	UGG​AGG​AGG​UGU​UGC​AGG​A Belmonte-Reche et al. (2020)	19	(+)	3466	Folded	(+)	40/44 (295 nm)	YES	YES
3	S2-3	GGG​UCA​GGG​UUU​AAA​UGG​U	19	(+)	4254	Folded	(+)	35/55 (295 nm)	YES	YES
4	S2-4	AGG​GUG​UGG​UUG​AUU​AUG​GUG	21	(+)	4503	Folded	(+)	28/33 (295 nm)	YES	YES
5	S2-5	UGG​AUC​UGG​GUA​AGG​AAG​GU	20	(−)	15921	nd	nd	42 (260 nm)	nd	NO (hp)
6	S2-6	GGG​GUG​CAU​UUC​GCU​GAU​UUU​GGG​G Bartas et al. (2020a)	25	(−)	28289	Folded	(−)	40 (260 nm)	nd	NO (hp)
7	S2-7	UGGCUGGCAAUGGCGGU Zhao et al. (2021), Belmonte-Reche et al. (2020)	17	(+)	28903	Folded	(−)	58 (260 nm)	nd	NO (hp)
SARS-CoV (GCF_000864885.1)										
1	S1-1	GUG​GCU​UCG​GGG​ACU​CUG​UGG​AAG​AGG​C	28	(+)	349	Folded	(-)	65 (260 nm)	nd	NO (hp)
2	S1-2	AGG​GAG​GUA​GGU​UUG​UGC​UGG	21	(+)	12721	Folded	(+)	41 (295 nm)	YES	YES
3	S1-3	UGU​GGG​AAG​GAC​AUA​AGG​UGG​UA	23	(−)	24586	Folded	(+)	25/50 (295 nm) 49 (260 nm)	nd	YES (G4<->hp)
4	S1-4	UGC​GGG​GCU​GCU​UGU​GGG​AAG​GA	23	(−)	24575	Folded	(+)	42 (295 nm)	YES	YES
5	S1-5	UGGGUGACUGGCGGGA	16	(+)	26611	Folded	(+)	45/55 (295 nm)	YES	YES
6	S1-6	UGG​AGG​ACG​CAA​UGG​GGC​AAG​GC	23	(+)	28204	Folded	(+)	35/40 (295 nm) 55 (260 nm)	nd	YES (G4<->hp)
7	S1-7	UGG​GUA​AAC​CUU​GGG​GUC​GGC​GCU​GUU​UUG​GC	32	(−)	28229	Folded	(−)	47 (260 nm)	nd	NO (hp)
MERS-CoV (GCF_000901155.1)										
1	M-1	GGG​UUU​GCC​UGU​GGA​UGU​GGG​G	22	(+)	1337	Folded	(+)	42/56 (295 nm) 50/53 (260 nm)	nd	YES (G4<->hp)
2	M-2	GGG​UUU​GUG​GUG​GUC​AAU​GG	20	(+)	2345	Folded	(+)	46/56 (295 nm)	YES	YES
3	M-3	GUG​GAA​GAU​GGU​UGU​GUG​UG	22	(+)	5020	Folded	(+)	38 (295 nm)	YES	YES
4	M-4	AGGGGGCUGCGUGGC	15	(+)	18461	Folded	(−)	47/52 (260 nm)	nd	NO (hp)
5	M-5	AGAGGAGGAGGGAGGU	16	(−)	25104	Folded	(+)	63 (295 nm)	YES	YES
6	M-6	UGG​GAC​UAG​CUG​GAC​GGG​A	19	(−)	26865	Folded	(−)	40 (260 nm)	nd	NO
7	M-7	GGG​UUU​GUG​GUG​GUC​AAU​GG	20	(−)	27807	Folded	(+)	61/79 (295 nm)	YES	YES
8	M-8	AGG​UGG​AAA​GGU​AAG​AGG​GAG	21	(−)	28721	Folded	(+)	36/44 (295 nm)	YES	YES
9	M-9	UGG​GUA​UUG​GUG​GAG​ACA​GGA	21	(+)	28789	Folded	(−)	25 (260 nm)	nd	NO
10	M-10	AGGGGACUGGAGGC	14	(+)	29045	Folded	(+)	41/59 (295 nm)	YES	YES

^a^ Sequence retrieved from the reference isolate for each virus.

^b^ CD: Circular Dichroism. Folded/unfolded depending on the intensity of the CD spectra. nd: not determined.

^c^ TDS: Thermal differential spectrum. (+) indicates the presence of a peak at 295 nm. (−) indicate the absence of the peak.

^d^ T_m_: Thermal melting temperature (the standard error is of ±1°C). Two T_m_ values are indicated when the melting process presents an hysteresis. The first value is for the cooling temperature ramp and the second value is for the heating.

^e^ NMR: Yes indicates the presence of imino proton resonances. No indicates the absence of imino protons resonances.

^f^ G4? Yes or No indicate that the sequence forms or not a G4 according to the data of CD, TDS, T_m_ and NMR. Hp means that a RNA hairpin structure is predicted by VIENNA software. G4<->hp: we speculate a G4/Hairpin equilibrium.

### Analysis of putative G4 forming sequences by circular dichroism spectroscopy

**FIGURE 3:**
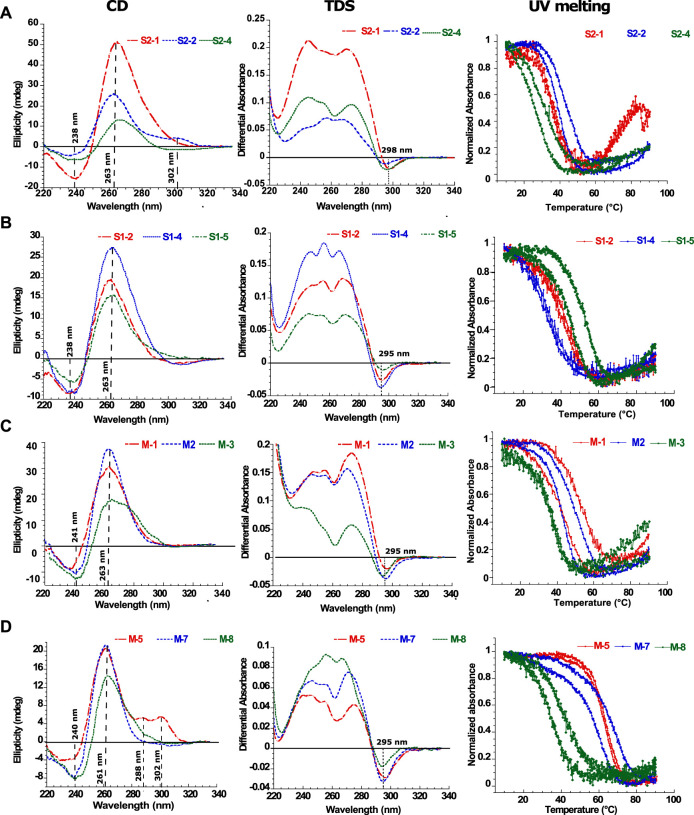
Biophysical analysis of the putative G4-forming sequences. Examples of *in vitro* characterization of putative G4s by Circular Dichroism (CD), Thermal Differential Spectra (TDS) and Thermal melting experiments measured at 295 nm (T_m_) for **(A)** SARS-CoV-2, **(B)** SARS-CoV and **(C,D)** MERS-CoV. For each UV-melting experiments the heating and cooling profiles are presented. All experiments were performed at around 4 µM RNA strand concentration dissolved in 10 mM lithium cacodylate pH 7 and 120 mM KCl. The results for the remaining 12 sequences are presented in the supplementary materials (Supplementary Figures S4–S27).

Circular Dichroism (CD) spectroscopy is a suitable tool to study the folding of nucleic acid structures. In the case of DNA structures, this technique successfully assigns specific CD spectra signatures to a given DNA topology including B-DNA, G4s or I-motifs (Villar-Guerra et al., 2018). In the case of RNA structures, CD spectra unfortunately show less discrimination. For instance, a stem-loop (or hairpin) RNA structure will give a similar spectral signature as for a G4 with a minimum at 245 nm and a maximum at 264 nm. However, since the CD signal is very sensitive to base stacking, it will still give valuable information on whether the structure is folded or unfolded. The CD spectra are summarized in the first column in Figure 3 for selected G4FS, and presented in detail for all tested G4FS in Supplementary Figures S4D–S27D. All of the 24 sequences tested display similar CD spectra with a single positive peak at 264 nm that indicates significant base stacking and suggests the formation of RNA secondary structures. Some sequences (S2-2 and M-5) also display a weaker positive peak at 290 nm which is not typically observed in RNA structures.

### Analysis of putative G4 forming sequences by thermal differential spectra

Thermal Differential Spectra (TDS) are obtained by a simple arithmetic subtraction of the absorbance spectrum of the sequence at a high temperature (unfolded) state from that of the low temperature (folded) state (Mergny, 2005). This technique enables the identification of various nucleic acid structures such as duplexes, triplexes, i-motifs or G4s. For example, the observation of a negative peak at 295 nm would reveal the presence of a G4 forming sequence, whereas this peak is absent for hairpin duplexes. The second column of Figure 3 shows examples of G4-forming sequences from the three viruses. These spectra present a negative minimum at 297 nm and two or three positive maxima between 246 and 272 nm. These features are consistent with the formation of G4 structures. Conversely, in the case of S2-6 (Supplementary Figure S9), S2-7 (Supplementary Figure S10) S1-1 (Supplementary Figure S11), S1-7 (Supplementary Figure S17), M-4 (Supplementary Figure S21), M-6 (Supplementary Figure S23) and M-9 (Supplementary Figure S26) the thermal difference spectra are positive with a minimum or approximately 225 nm and a maximum around 270 nm. The missing negative minima around 295 nm argues for the absence of G4s.

### Analysis of putative G4 forming sequences by UV-melting

UV-melting experiments record the cooperative transition between the folded and unfolded forms of a given oligonucleotide. At 295 nm, a UV-melting-curve of a G4 structure usually results in a hypo-chromic (decrease of absorbance) melting process presenting a cooperative sigmoidal transition (Mergny and Lacroix, 2009). For instance, S2-1, S1-2, S1-4, M-3 and M-5 oligonucleotides present a reversible transition because the heating and cooling processes are superimposed. This reversibility indicates the folding and unfolding kinetics of the structure are relatively fast which strongly suggest the formation of a mono-molecular (formed by one strand) G4 structure (Mergny, 2005). The UV-melting experiments also allows us to estimate the stability of the G4 structures by measuring the melting temperature (T_m_): 40°C for S2-1, 41°C for S1-2, 42°C for S1-4, 38°C for M-3 and 63°C for M-5. However, in most cases, the melting process is not reversible and presents a hysteresis (the heating and cooling profiles have two different half transitions: T_1/2_), indicating relatively slow folding and unfolding kinetics. Important hysteresis (for instance 4°C for S2-2 and 25°C for S1-3) results from slower folding kinetics due to the formation of multimeric G4s (formed by two or more strands). This is observed for S1-3 (Supplementary Figure S13), S1-5 and S1-6 (Supplementary Figures S15, S16), M-1, M-2 (Supplementary Figures S19–S21), M-7, M-8 and M-10 (Supplementary Figures S24–S27) and also S2-2, S2-3, S2-4 (Supplementary Figures S5–S7). In the case of the non-G4-forming sequences S2-5, S2-6, S2-7 (Supplementary Figures S8–S10), S1-1 (Supplementary Figure S11), S1-7 (Supplementary Figure S17), M-4 (Supplementary Figure S21), M-6 (Supplementary Figure S23) and M-9 (Supplementary Figure S26), the melting profiles at 295 nm do not present any hypo-chromism and no structural transitions are detected. However, at 260 nm the processes are reversible with no hysteresis suggesting the formation of stable mono-molecular secondary structures, very likely hairpins as predicted by RNAfold in some cases (Supplementary Figures S8–S11, S13, S16–S18, S21) with T_m_ ranging from 25°C to 65°C. In particular, S1-3, S1-5 and M1 show distinct behavior. At 260 nm, the melting profiles are reversible, whereas at 295 nm, a significant hysteresis is present. We interpret this behavior as an equilibrium between a multi-molecular G4 and a hairpin-like structure.

### Analysis of putative G4 forming sequences by ^1^H-1D NMR

**FIGURE 4:**
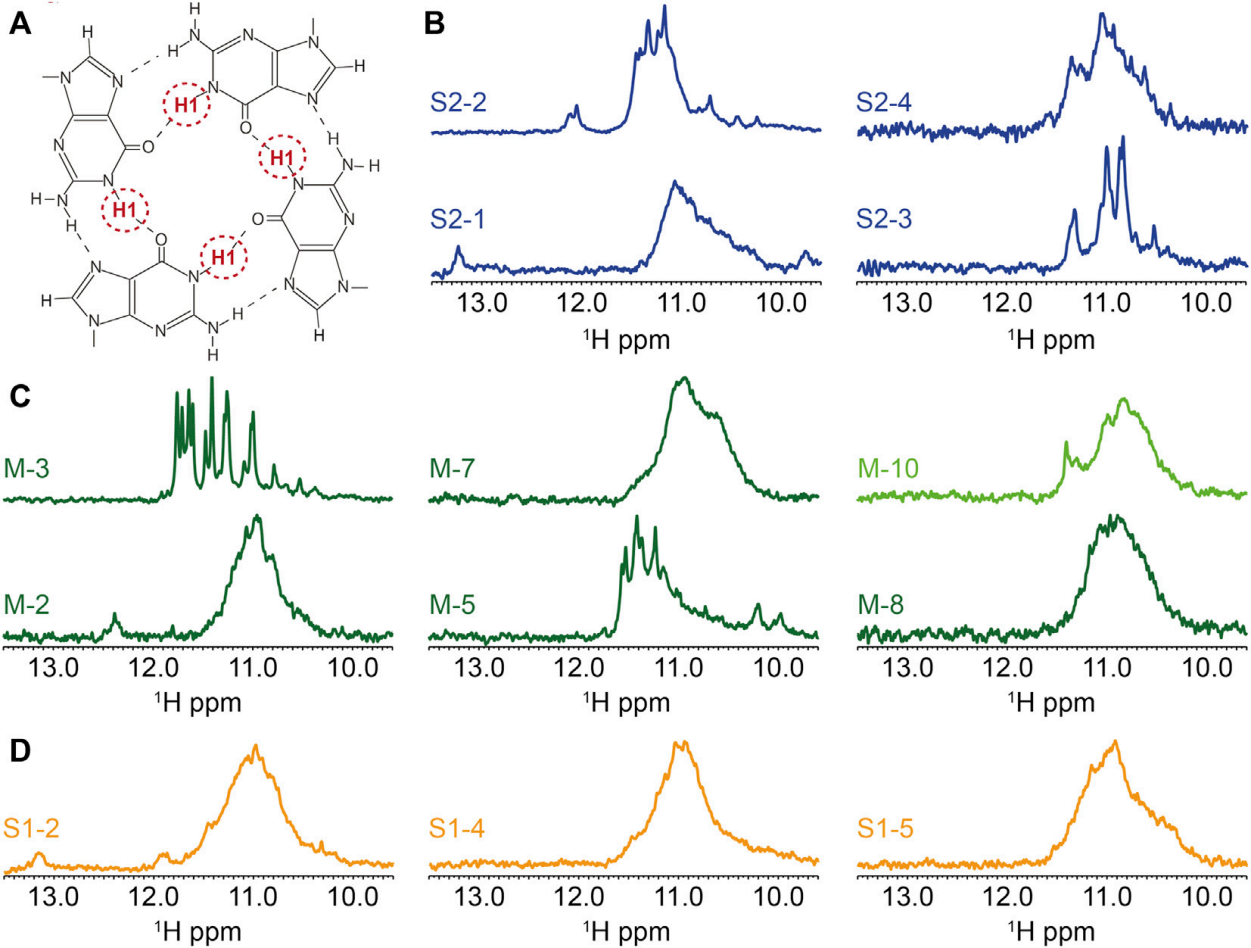
Biophysical analysis of the putative G4-forming sequences by ^1^H-1D NMR. **(A)** Representation of the imino proton involved in the formation of G-tetrads and detected by ^1^H-1D NMR. ^1^H-1D NMR experiments depicting the imino region for **(B)** SARS-CoV-2, **(C)** MERS-CoV and **(D)** SARS-CoV. All experiments were performed at around 100 µM RNA strand concentration dissolved in 20 mM potassium phosphate pH7 and 120 mM KCl at 15°C in 90% H2O/10%. M-10 was recorded at 4°C.

The final characterization to confirm the formation of G4 structures used ^1^H-1D NMR experiments. The imino proton H1 from guanine (Figure 4A), or H3 in uracil, can normally only be observed by NMR when they are involved in hydrogen-bonds or are otherwise protected from exchanging with water molecules. Furthermore, their chemical shift is indicative of the type of base-paring involved (Wang et al., 2021). For standard base pairs, a GC guanine H1 and a UA uracil H3 have peaks at [12–13] ppm and [13–14] ppm, respectively. In contrast, the four imino H1 protons from guanines involved in an RNA G-tetrad corresponds to four peaks in the region from [10–12] ppm. However, it should also be noted that the guanine H1 and uracil H3 protons in GU or GG base-pairs will also present two peaks in the same region as the G-tetrad.

We present the ^1^H-1D NMR spectra for a selection of G4FS in Figure 4. In the case of M-3, the presence of a set of well resolved peaks in the [10–12] ppm region confirms the formation of G4s, with a limited conformational polymorphism that is suggested by a second set of minor peaks. The presence of 12 major peaks also suggests the formation of a three-tetrad G-quadruplex. M-5, S2-4, S2-2 and S2-3 (Figures 4B–D) also exhibit G4 signatures with quite well resolved peaks in the [10–12] ppm region but the signal broadening indicate a higher polymorphism. S2-1, M-2, M-7, M-8, S1-2, S1-4 and S1-5 present a very broad NMR signal in the [10–12] ppm suggesting the formation of highly polymorphic G4 structures. Finally, very weak peaks were detected in the case of M10, indicating an aggregation or that only a small fraction of the sequences forms a G4. Peaks of higher intensity only appeared when the spectrum was recorded at 4°C.

### Monomeric G4s are mainly located in the ORF1A and S genes

**FIGURE 5:**
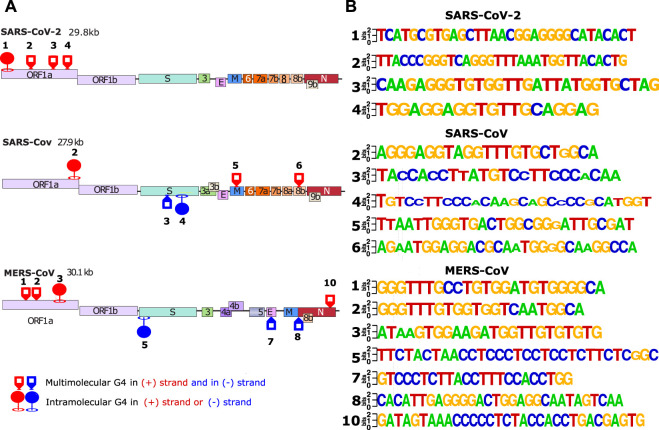
*In vitro* validated and conserved G4-forming sequences. **(A)** The location of the validated G-quadrupexes in the 3 different viral genomes (SARS-CoV-2, SARS-CoV and MERS-CoV). The validated G4s were categorized by 2 different criterias: forming intra/multi-molecular G4s. **(B)** LOGO representation of the validated G4s made with weblogo.berkeley.edu. For the logo representation, alignments have been made in cluster type on all SARS-2 (2500 sequences), SARS (308 sequences) and MERS (459 sequences) complete genomes available on the NCBI.

The results of the biophysical analysis of the 24 putative G4FS found in the three coronaviruses are summarized in Table 1 and Figure 5. By combining the results from the separate techniques, we found that 16 sequences were most likely to form G4 structures (Figure 5A), with 8 forming other secondary structures like hairpins. The 16 G4s are all highly conserved as suggested by the logo representations of the sequence alignments (Figure 5B). Among the G4s, 11 sequences form multi-molecular G4s and 5 form mono-molecular G4s. Interestingly, mono-molecular G4s are mainly present in ORF1A of the three viruses and S genes of SARS-1 and MERS.

## Discussion

In this study, we wanted to contribute to the worldwide research effort to fight coronaviruses and in particular SARS-CoV-2. Since RNA G4s are becoming a promising antiviral target (Abiri et al., 2021), (Ruggiero and Richter, 2018), we wanted to investigate if G4s are formed in SARS-CoV-2, SARS-CoV-1 and MERS-CoV. In previous investigations, this effort has mainly been carried out through bioinformatics analyses (Zhao et al., 2021)^,^ (Belmonte-Reche et al., 2020), (Bartas et al., 2020b) that could lead to the detection of false positives. In our approach, we believed that it was necessary to confront the predictions made *in silico* with an *in vitro* validation by biophysical methods. Indeed, we found that among the 24 sequences that we predicted and tested, only 5 were actually forming stable mono-molecular G4s. The biophysical validation of the predictions is indeed important; however, it is also essential to use separate biophysical techniques to obtain reliable conclusions. In fact, Circular Dichroism alone cannot distinguish a G4 RNA structure from a hairpin RNA because they both present a maximum around 260 nm and a minimum around 240 nm. The circular dichroism analysis in combination with the TDS and UV-melting techniques allows to better discriminate the two structures. For instance, the sequences S2-6 (Bartas et al., 2020b) and S2-7 (Belmonte-Reche et al., 2020) (Zhao et al., 2021) have been presented in other investigations as forming G4s. In our analysis they do not form G4s but hairpin structures instead. Moreover, in our approach we have stressed the reversibility of the thermal transition obtained by UV-melting analysis. This allowed us to get an idea of the kinetics of structure formation and therefore to have an insight on its molecularity. Indeed, the presence of an hysteresis in the melting profile argue for the presence of a multi-molecular form. In most previous studies on SARS-CoV-2 G4s, only thermal denaturation ramps (heating) were performed/shown and not renaturations (cooling), thus missing the information on the molecularity. Here, we found that the majority of the G4 structures detected in SARS-CoV-2 (11/16) were not mono-molecular, but multi-molecular which is a crucial information in the case of G4 RNA structures.

Notably the RNA G4s we validated are mostly present in the ORF1A gene of the positive strand and in the negative RNA strand of the S gene. As a result, the three coronavirus genomes show a significantly reduced frequency of G4 motifs. This trend has also been observed in several bioinformatics studies. For instance, G4-hunter algorithm has previously been applied to detect putative G4FS in several Nidoviral genomes (Bartas et al., 2020b). The highest frequency was obtained in Arteriviridae and the lowest in Coronaviridae. SARS-CoV-2 genome showed the most pronounced scarcity of G4s with a putative G4FS density in the lower part of the Coronaviridae family (Belmonte-Reche et al., 2021). Conversely, SARS-CoV-2 genome is rich in inverted repeats that can adopt RNA hairpins or cruciform structures (Goswami et al., 2021). These structural motifs are important for proper genome organization and are often specifically recognized by regulatory proteins. Hence, G4s have been effectively eliminated during evolution in order to favor hairpins or cruciform structures. The existence of these very rare but conserved G4 structures, despite this negative constraint, highlights their importance.

**FIGURE 6:**
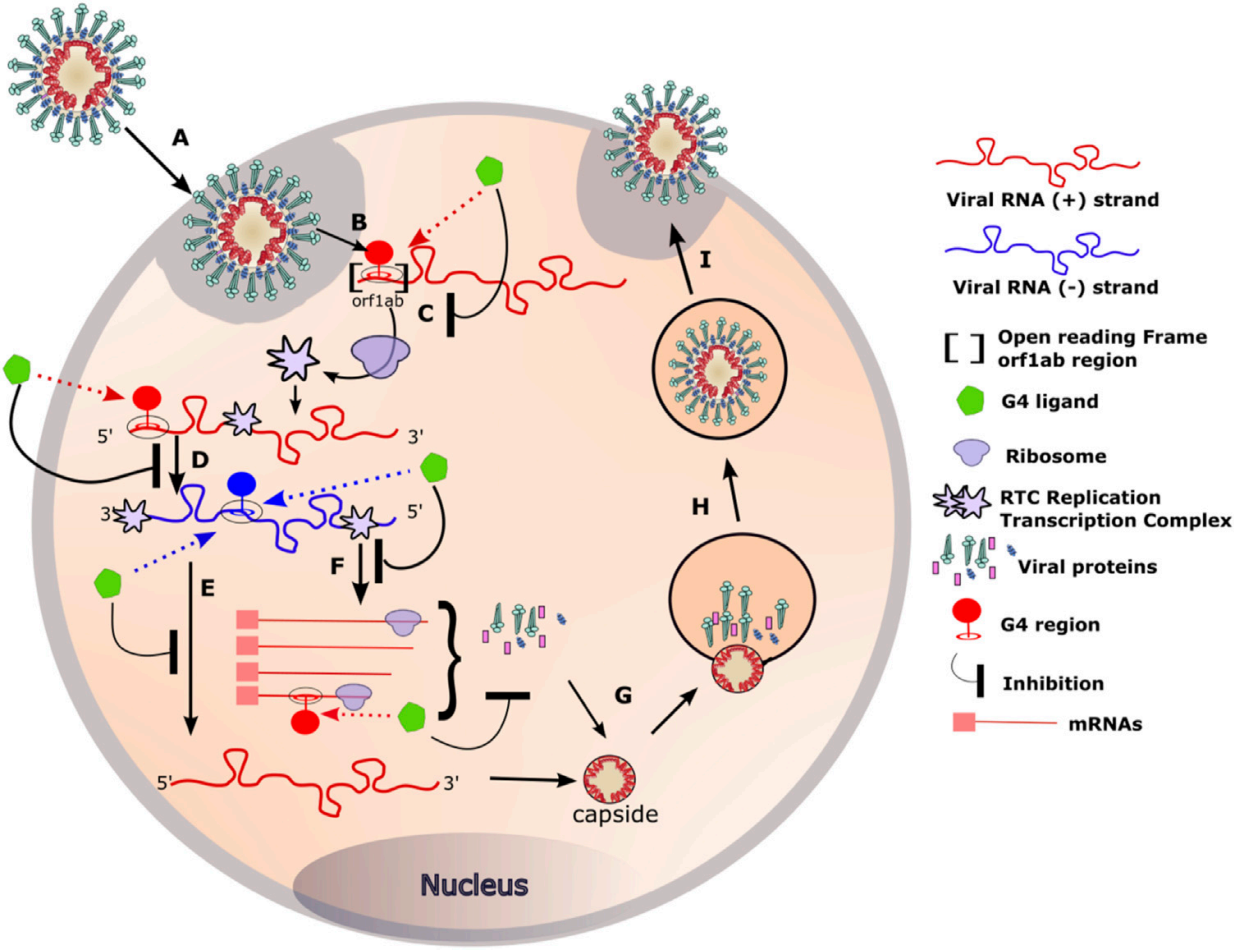
RNA G4 formation during coronavirus replication and targeting by G4 ligands. **(A)** Coronaviruses bind, *via* the S protein (for Spike), to the ACE2 receptor expressed on the surface of the target cells. The viral particle penetrates the cells by endocytosis or by direct fusion to the plasma membrane. **(B)** Release of the genomic RNA in the cytoplasm. **(C)** The open reading frame (Orf1ab) present in the RNA with positive (+) polarity (in red) is translated to produce the viral replication and transcription complex (RTC). **(D)** The RTC will transcribe the (+) viral RNA (red) into its complementary viral RNA with negative (−) polarity (in blue). **(E)** The (−) RNA will serve as a template for the synthesis of the complete (+) RNA that will be incorporated in the viral particle. **(F)** The (−) RNA will also serve as a template for the synthesis of mRNA that will be translated into the structural viral proteins. **(G)** Genomic RNA and structural proteins will then assemble in the endoplasmic reticulum. **(H, I)** The newly formed viruses are then transported *via* transport vesicles to the cell surface, where they are released. The presence of G-quadruplexes in both the negative and positive strands of the viral genome can be targeted by G4 ligands at different stages of the viral cycle. Binding of the G4 ligands may inhibit the replication of the virus inside the cell.

Hence, targeting these G4s and stabilizing them with small molecules could alter viral fitness. Indeed, two different studies have recently demonstrated SARS-CoV-2 inhibition by two well-known G4-ligands: PDS and TMPyP4. PDS compound, a G4-specific stabilizer, has been shown to attenuate SARS-CoV-2 infectivity in both pseudovirus cell systems and mouse models (Liu et al., 2022). More recently, TMPyP4 G4-ligands, already known for its antiviral activities against HIV-1, also exhibited antiviral activity against SARS-CoV-2. Remarkably, in Syrian hamster and transgenic mouse models of SARS-CoV-2 infection, administration of TMPyP4 resulted in a significant reduction in viral loads and lung injury (Qin et al., 2022). In the same study, the activity of TMPyP4 proved to be significantly stronger than that of remdesivir. These findings highlight the high potential of an antiviral strategy based on targeting G4 RNAs present in the SARS-CoV-2 genome. Inhibition mechanism is still unknown but it may be mediated by various mechanisms, as shown in Figure 6.

### Targeting ORF1a RNA G4s during translation

Besides defining the amino acid sequence of proteins, an mRNA can also regulate gene expression through the formation of RNA secondary structures (Ding et al., 2014). These structures can have a direct impact on protein translation. Several studies have already proposed novel mechanisms of G4-mediated gene regulation at the translational level. For instance, RNA G4s can act as obstacles to the progression of ribosomes on their mRNA templates (Endoh and Sugimoto, 2016). Furthermore, depending on its stability, an RNA G4 located in an open reading frame can also inhibit protein expression (Endoh et al., 2013) by promoting termination and/or ribosome stalling. In the case of Epstein Barr virus, the stabilization of RNA G4s by G4 ligands (pyridostatin derivatives) slows down the translation of viral mRNA into maintenance proteins (Murat et al., 2014).

Ribosomal frame-shifting is a well-known re-encoding process that occurs during translation elongation and is stimulated by secondary mRNA structures such as pseudo-knot stem-loop or G4s (Yu et al., 2014), (Howard et al., 2004). For instance, Ribosomal -1-frameshift (-1-FS) is a translational mechanism whereby ribosomes are forced to move back one nucleotide, leading to the decoding of a second open reading frame (ORF). It has been shown that G4 RNAs are able to stimulate this displacement *in vitro* and in cultured cells (Yu et al., 2014). The efficiency of the displacement is directly correlated with the stability of the G4 structure. Indeed, the stabilization of the G4 by PhenDC3 or PDS (Zhao et al., 2021) G4 ligand, induced an increase of the displacement rates. It has also been proposed that ribosomal frame-shift is stimulated by RNA dimerisation through the formation of kissing complexes in SARS-CoV (Ishimaru et al., 2013). Viral RNA dimerization is also possible *via* the formation of dimeric G4 structures as has already been proposed in HIV-1 (Shen et al., 2011). Such dimeric G4 intermediates could also be formed in SARS-CoV-2.

One of the most important steps of the coronaviruses replication cycle is the primary translation of ORF1AB (Figure 6 step C). It occurs in the early steps of the infection and results in the production of the RTC: the viral replication and transcription complex. A properly produced RTC is essential for the correct functioning of all downstream stages of the virus cycle. In our opinion, altering ribosomal progression by G4 ligands or changing the ORF could lead to deleterious consequences for the virus.

### Targeting RNA G4s during RNA transcription

G4s not only act as roadblocks for ribosomes, but also perturb the progression of RNA polymerases and reverse transcriptases on their RNA template. For instance, in the case of HSV-1, *in vitro* assays revealed the ability of G4 ligands to stabilise HSV-1 G4s and block polymerase progression (Callegaro et al., 2017). Likewise, the stalling of viral RNA-dependent RNA polymerase, induced by the G4 ligands TMPyP4 and a PDS derivative (PDP), resulted in a decrease of HCV core protein expression (Wang et al., 2016). In the case of coronaviruses, such stalling could occur in the step D, E or F of the viral cycle (Figure 6).

Several RNA binding proteins like hnRNP A1 or nucleolin have been shown to interact with G4s and some of them are involved in the replication of other viruses such as HCV (Bian et al., 2019), (Lista et al., 2017). Among the 16 non-structural proteins (Nsp1-16) encoded by ORF1a and ORF1b, Nsp3 protein is involved in the assembly of the viral RTC (Lei et al., 2018). NSP3 contains a domain called SUD, that is conserved in SARS-1 and SARS-2 and is responsible of the high pathogenicity of these two viruses. Interestingly, the SUD domain is able to interact with DNA and RNA G4s (Lavigne et al., 2021). Therefore, binding of NSP3 to the RNA G4s present in the viral genome may occur during the early steps of the viral cycle. Disrupting this interaction using G4 ligands can affect the correct progression of the replication cycle.

We have identified 5 RNA G4s in SARS-1, SARS-2 and MERS that are mostly present in ORF1A gene. The presence of these G4 structures, despite an overall negative evolutionary constraint, highlights their importance. Targeting these RNA structures may represent a promising new antiviral avenue with limited risks of developing resistance as suggested by the high conservation of the viral G4s.

## Materials and methods

### Bioinformatics detection of putative G4 forming sequences

Several programs were used to detect the presence of guanine rich sequences that are likely to form G-quadruplexes. In the first step, Seaview software (Galtier et al., 1996) was used for the sequence visualization and alignment. Alignments have been made on all SARS-CoV-2 (2500 sequences), SARS-CoV (308 sequences) and MERS-CoV complete genomes (459 sequences) available on the National Center for Biotechnology Information [https://www.ncbi.nlm.nih.gov/]. From the alignments, the G4hunter software (Bedrat et al., 2016) was used to scan the alignment and detect sequences that can form G4 structures. G4H is a scoring algorithm that assigns each nucleotide with a score over a chosen window (20 in this study). A positive score is given for the Guanines and a negative for the Cytosines, whereas the other bases have a score of 0. The G4H score is then calculated for each 20 nucleotides window. Then an average of all the G4H scores of the alignment is calculated. To select the putative G4 forming sequences we chose to use a threshold of 1 (in absolute value) which is a good compromise between sensitivity and accuracy. The positive scores will predict G4FS that are present in the (+) strand while the negative scores will predict G4FS in the (-) strand. From the identified sequences, the WebLogo software (http://weblogo.berkeley.edu) (Crooks et al., 2004), (Schneider and Stephens, 1990) was used to visualize the conservation. In addition, the possibility of RNA structures other than G4s were predicted by using RNAfold (Gruber et al., 2008) available on the Vienna RNA Websuite (http://rna.tbi.univie.ac.at/cgi-bin/RNAWebSuite/RNAfold.cgi **):** RNA secondary structure predictions were done on the Vienna RNA Websuite.

### Oligonucleotides Synthesis

Oligonucleotides Synthesis: Oligonucleotide were synthesized in the lab according to the standard beta-phosphoramidite methodology using an H8 automated synthesizer with. The DMT protection group was left on the 5’ terminus. Poly-pak purification procedure was performed to de-protect the synthetic oligonucleotides using poly-pak 2 cartridges. Oligonucleotide strand concentrations were determined by measuring absorbance at 260 nm with the extinction coefficient determined by an algorithm based on the nearest neighbour method using the IDT oligo calc online software.

### Circular dichroism spectroscopy

CD spectra were measured in 10 mm path-length quartz cells using a (Jasco J-1500 instrument). Each final spectrum was the average of two scans between 220 and 335 nm at a rate of 50 nm/min, with 0.5 nm data pitch, 2 nm bandwidth, and 2 s response time. Spectra were measured in 10 mm path-length quartz cells with a 4 µM strand concentration in a buffer composed of 10 mM lithium cacodylate (pH 7.0) and 120 mM KCl. Spectra were recorded at 20°C, 15°C or 4°C.

### Ultraviolet melting measurements and thermal difference spectra

UV melting assays were measured on a SAFAS UVmc2 double beam spectrophotometer equipped with a 10-cell holder and Peltier controller. Profiles were measured in 10 mm path-length quartz cells at the wavelengths of 260, 273 and 295 nm, with a 4 µM strand concentration in a buffer composed of 10 mM lithium cacodylate (pH 7.0) and 120 mM KCl. The heating and cooling curves were recorded between 90°C and 10°C with a temperature gradient of 0.4°C/min. Thermal difference spectra TDS were calculated by subtracting the UV spectrum at 10°C from the UV spectrum at 90°C.

### NMR experiments

A 700 MHz Bruker spectrometer was used to perform the NMR experiments. Sample strand concentrations was around 100 µM dissolved in 20 mM potassium phosphate pH 7 and 120 mM KCl in 90% H2O/10% in 5 mm tubes. The 1D-1H-NMR spectra were recorded using a double pulse field gradient perfect spin echo (zgesgppe) pulse sequence to suppress the water signal. Spectra were recorded with a spectral width of 19 ppm, an acquisition time of 1.2s and a relaxation delay of 2s. Experiments were performed at 15°C and 4°C.

## Data Availability

The original contributions presented in the study are included in the article/Supplementary Material; further inquiries can be directed to the corresponding author.

## References

[B1] Abiri A. Lavigne M. Rezaei M. Nikzad S. Zare P. Mergny J-L. (2021). “Unlocking G-quadruplexes as antiviral targets,” Pharmacol. Rev. Touyz, 73, 897–923. 10.1124/pharmrev.120.000230 34045305

[B2] Amrane S. Kerkour A. Bedrat A. Vialet B. Andreola M. L. Mergny J. L. (2014). Topology of a DNA G-quadruplex structure formed in the HIV-1 promoter: A potential target for anti-HIV drug development. J. Am. Chem. Soc. 136 (14), 5249–5252. 10.1021/ja501500c 24649937

[B3] Bartas M. Brazda V. Bohalova N. Cantara A. Volna A. Stachurova T. (2020b). In-Depth bioinformatic analyses of nidovirales including human SARS-CoV-2, SARS-CoV, MERS-CoV viruses suggest important roles of non-canonical nucleic acid structures in their lifecycles. Front. Microbiol. 11, 1583. 10.3389/fmicb.2020.01583 32719673 PMC7347907

[B4] Bartas M. Brazda V. Bohalova N. Cantara A. Volna A. Stachurova T. (2020a), In-depth bioinformatic analyses of human SARS-CoV-2, SARS-CoV, MERS-CoV, and other nidovirales suggest important roles of noncanonical nucleic acid structures in their lifecycles. preprint. Bioinformatics 10.1101/2020.04.09.031252 PMC7347907 32719673

[B5] Bedrat A. Lacroix L. Mergny J. L. (2016). Re-evaluation of G-quadruplex propensity with G4Hunter. Nucleic Acids Res. 44 (4), 1746–1759. 10.1093/nar/gkw006 26792894 PMC4770238

[B6] Belmonte-Reche E. Serrano-Chacón I. Gonzalez C. Gallo J. Bañobre-López M. (2020) Exploring G and C-quadruplex structures as potential targets against the severe acute respiratory syndrome coronavirus 2. preprint. Bioinformatics 10.1101/2020.08.19.257493

[B7] Belmonte-Reche E. Serrano-Chacón I. Gonzalez C. Gallo J. Bañobre-López M. (2021). Potential G-quadruplexes and i-Motifs in the SARS-CoV-2. PLOS ONE 16 (6), e0250654. 10.1371/journal.pone.0250654, 34101725 PMC8186786

[B8] Bian W.-X. Xie Y. Wang X. N. Xu G. H. Fu B. S. Li S. (2019). Binding of cellular nucleolin with the viral core RNA G-quadruplex structure suppresses HCV replication. Nucleic Acids Res. 47 (1), 56–68. 10.1093/nar/gky1177 30462330 PMC6326805

[B9] Biswas B. Kandpal M. Vivekanandan P. (2017). A G-quadruplex motif in an envelope gene promoter regulates transcription and virion secretion in HBV genotype B. Nucleic Acids Res. 45 (19), 11268–11280. 10.1093/nar/gkx823 28981800 PMC5737607

[B10] Brázda V. Bartas M. Bowater R. P. (2021). Evolution of diverse strategies for promoter regulation. Trends Genet. 37 (8), 730–744. 10.1016/j.tig.2021.04.003 33931265

[B11] Bryan T. M. (2020). G-quadruplexes at telomeres: Friend or foe? Molecules 25 (16), 3686. 10.3390/molecules25163686 32823549 PMC7464828

[B12] Callegaro S. Perrone R. Scalabrin M. Doria F. Palu G. Richter S. N. (2017). A core extended naphtalene diimide G-quadruplex ligand potently inhibits herpes simplex virus 1 replication. Sci. Rep. 7 (1), 2341. 10.1038/s41598-017-02667-3 28539620 PMC5443766

[B13] Cammas A. Millevoi S. (2016). RNA G-quadruplexes: Emerging mechanisms in disease. Nucleic Acids Res. 45 (4), 1584–1595. 10.1093/nar/gkw1280 PMC5389700 28013268

[B14] Crooks G. E. Hon G. Chandonia J. M. Brenner S. E. (2004). WebLogo: A sequence logo generator: Figure 1. Genome Res. 14 (6), 1188–1190. 10.1101/gr.849004 15173120 PMC419797

[B15] Cui J. Li F. Shi Z.-L. (2019). Origin and evolution of pathogenic coronaviruses. Nat. Rev. Microbiol. 17 (3), 181–192. 10.1038/s41579-018-0118-9 30531947 PMC7097006

[B16] de Groot R. J. Baker S. C. Baric R. S. Brown C. S. Drosten C. Enjuanes L. (2013). Commentary: Middle East respiratory syndrome coronavirus (MERS-CoV): Announcement of the coronavirus study group. J. Virol. 87 (14), 7790–7792. 10.1128/JVI.01244-13 23678167 PMC3700179

[B17] de Wit E. Feldmann F. Cronin J. Jordan R. Okumura A. Thomas T. (2020). Prophylactic and therapeutic remdesivir (GS-5734) treatment in the rhesus macaque model of MERS-CoV infection. Proc. Natl. Acad. Sci. U. S. A. 117 (12), 6771–6776. 10.1073/pnas.1922083117 32054787 PMC7104368

[B18] Ding Y. Tang Y. Kwok C. K. Zhang Y. Bevilacqua P. C. Assmann S. M. (2014). *In vivo* genome-wide profiling of RNA secondary structure reveals novel regulatory features. Nature 505 (7485), 696–700. 10.1038/nature12756 24270811

[B19] Dong L. Hu S. Gao J. (2020). Discovering drugs to treat coronavirus disease 2019 (COVID-19). Drug Discov. Ther. 14 (1), 58–60. 10.5582/ddt.2020.01012 32147628

[B20] Endoh T. Kawasaki Y. Sugimoto N. (2013). Suppression of gene expression by G-quadruplexes in open reading frames depends on G-quadruplex stability. Angew. Chem. Int. Ed. 52 (21), 5522–5526. 10.1002/anie.201300058 23589400

[B21] Endoh T. Sugimoto N. (2016). Mechanical insights into ribosomal progression overcoming RNA G-quadruplex from periodical translation suppression in cells. Sci. Rep. 6 (1), 22719. 10.1038/srep22719 26948955 PMC4780275

[B22] Firth A. E. (2014). Mapping overlapping functional elements embedded within the protein-coding regions of RNA viruses. Nucleic Acids Res. 42 (20), 12425–12439. 10.1093/nar/gku981 25326325 PMC4227794

[B23] Galtier N. Gouy M. Gautier C. (1996). SEAVIEW and PHYLO_WIN: Two graphic tools for sequence alignment and molecular phylogeny. Bioinformatics 12 (6), 543–548. 10.1093/bioinformatics/12.6.543 9021275

[B24] Ganesh B. Rajakumar T. Malathi M. Manikandan N. Nagaraj J. Santhakumar A. (2021). Epidemiology and pathobiology of SARS-CoV-2 (COVID-19) in comparison with SARS, MERS: An updated overview of current knowledge and future perspectives. Clin. Epidemiol. Glob. Health 10, 100694. 10.1016/j.cegh.2020.100694 33462564 PMC7806455

[B25] Goswami P. Bartas M. Lexa M. Bohalova N. Volna A. Cerven J. (2021). SARS-CoV-2 hot-spot mutations are significantly enriched within inverted repeats and CpG island loci. Briefings Bioinforma. 22 (2), 1338–1345. 10.1093/bib/bbaa385 PMC7799342 33341900

[B26] Gruber A. R. Trent J. O. Chaires J. B. (2008). The Vienna RNA Websuite’, Nucleic Acids Research, 36(Web Server), pp. W70–W74. del Villar-Guerra. Angew. Chem. Int. Ed. 57 (24), 7171–7175. 10.1093/nar/gkn18810.1002/anie.201709184 PMC2447809 18424795

[B27] Howard M. T. Gesteland R. F. Atkins J. F. (2004). Efficient stimulation of site-specific ribosome frameshifting by antisense oligonucleotides. RNA 10 (10), 1653–1661. 10.1261/rna.7810204 15383681 PMC1370650

[B28] Ishimaru D. Plant E. P. Sims A. C. Yount B. L. Roth B. M. Eldho N. V. (2013). RNA dimerization plays a role in ribosomal frameshifting of the SARS coronavirus. Nucleic Acids Res. 41 (4), 2594–2608. 10.1093/nar/gks1361 23275571 PMC3575852

[B29] Jana J. Mohr S. Vianney Y. M. Weisz K. (2021). Structural motifs and intramolecular interactions in non-canonical G-quadruplexes. RSC Chem. Biol. 2 (2), 338–353. 10.1039/D0CB00211A 34458788 PMC8341446

[B30] Jaubert C. Bedrat A. Bartolucci L. Di Primo C. Ventura M. Mergny J. L. (2018). RNA synthesis is modulated by G-quadruplex formation in Hepatitis C virus negative RNA strand. Sci. Rep. 8 (1), 8120. 10.1038/s41598-018-26582-3 29802381 PMC5970142

[B31] Kabinger F. Stiller C. Schmitzova J. Dienemann C. Kokic G. Hillen H. S. (2021). Mechanism of molnupiravir-induced SARS-CoV-2 mutagenesis. Nat. Struct. Mol. Biol. 28 (9), 740–746. 10.1038/s41594-021-00651-0 34381216 PMC8437801

[B32] Kiening M. Ochsenreiter R. Hellinger H-J. Rattei T. Hofacker I. Frishman D. (2019). Conserved secondary structures in viral mRNAs. Viruses 11 (5), 401. 10.3390/v11050401 31035717 PMC6563262

[B33] Ksiazek T. G. Erdman D. Goldsmith C. S. Zaki S. R. Peret T. Emery S. (2003). A novel coronavirus associated with severe acute respiratory syndrome. N. Engl. J. Med. Overseas. Ed. 348 (20), 1953–1966. 10.1056/NEJMoa030781 12690092

[B34] Lago S. Nadai M. Cernilogar F. M. Kazerani M. Dominiguez Moreno H. Schotta G. (2021). Promoter G-quadruplexes and transcription factors cooperate to shape the cell type-specific transcriptome. Nat. Commun. 12 (1), 3885. 10.1038/s41467-021-24198-2 34162892 PMC8222265

[B35] Lavigne M. Helynck O. Rigolet P. Boudria-Souilah R. Nowakowski M. Baron B. (2021). SARS-CoV-2 Nsp3 unique domain SUD interacts with guanine quadruplexes and G4-ligands inhibit this interaction. Nucleic Acids Res. 49 (13), 7695–7712. 10.1093/nar/gkab571 34232992 PMC8287907

[B36] Lei J. Kusov Y. Hilgenfeld R. (2018). Nsp3 of coronaviruses: Structures and functions of a large multi-domain protein. Antivir. Res. 149, 58–74. 10.1016/j.antiviral.2017.11.001 29128390 PMC7113668

[B37] Letko M. Marzi A. Munster V. (2020). Functional assessment of cell entry and receptor usage for SARS-CoV-2 and other lineage B betacoronaviruses. Nat. Microbiol. 5 (4), 562–569. 10.1038/s41564-020-0688-y 32094589 PMC7095430

[B38] Li Q. Guan X. Wu P. Wang X. Zhou L. Tong Y. (2020). Early transmission dynamics in wuhan, China, of novel coronavirus–infected pneumonia. N. Engl. J. Med. Overseas. Ed. 382 (13), 1199–1207. 10.1056/NEJMoa2001316 PMC7121484 31995857

[B39] Li Q. Xiang J. F. Yang Q. F. Sun H. X. Guan A. J. Tang Y. L. (2013). G4LDB: A database for discovering and studying G-quadruplex ligands. Nucleic Acids Res. 41 (D1), D1115–D1123. 10.1093/nar/gks1101 23161677 PMC3531060

[B40] Lista M. J. Martins R. P. Billant O. Contesse M. A. Findakly S. Pochard P. (2017). Nucleolin directly mediates Epstein-Barr virus immune evasion through binding to G-quadruplexes of EBNA1 mRNA. Nat. Commun. 8 (1), 16043. 10.1038/ncomms16043 28685753 PMC5504353

[B41] Liu D. X. Fung T. S. Chong K. K. L. Shukla A. Hilgenfeld R. (2014). Accessory proteins of SARS-CoV and other coronaviruses. Antivir. Res. 109, 97–109. 10.1016/j.antiviral.2014.06.013 24995382 PMC7113789

[B42] Liu G. Du W. Sang X. Tong Q. Wang Y. Chen G. (2022). RNA G-quadruplex in TMPRSS2 reduces SARS-CoV-2 infection. Nat. Commun. 13 (1), 1444. 10.1038/s41467-022-29135-5 35301316 PMC8931161

[B43] Mergny J.-L. (2005). Thermal difference spectra: A specific signature for nucleic acid structures. Nucleic Acids Res. 33 (16), e138. 10.1093/nar/gni134 16157860 PMC1201377

[B44] Mergny J. Lacroix L. (2009). UV melting of G‐quadruplexes. Curr. Protoc. Nucleic Acid. Chem. 37 (1), Unit 17.1. 10.1002/0471142700.nc1701s37 19488970

[B45] Métifiot M. Amrane S. Litvak S. Andreola M. L. (2014). G-Quadruplexes in viruses: Function and potential therapeutic applications. Nucleic Acids Res. 42 (20), 12352–12366. 10.1093/nar/gku999 25332402 PMC4227801

[B46] Murat P. Zhong J. Lekieffre L. Cowieson N. P. Clancy J. L. Preiss T. (2014). G-quadruplexes regulate Epstein-Barr virus–encoded nuclear antigen 1 mRNA translation. Nat. Chem. Biol. 10 (5), 358–364. 10.1038/nchembio.1479 24633353 PMC4188979

[B47] Onel B. Lin C. Yang D. (2014). DNA G-quadruplex and its potential as anticancer drug target. Sci. China Chem. 57 (12), 1605–1614. 10.1007/s11426-014-5235-3 27182219 PMC4863707

[B48] Owen D. R. Allerton C. M. N. Anderson A. S. Aschenbrenner L. Avery M. Berritt S. (2021). An oral SARS-CoV-2 Mpro inhibitor clinical candidate for the treatment of COVID-19. Science 374 (6575), 1586–1593. 10.1126/science.abl4784 34726479

[B49] Peiris J. S. M. Lai S. Poon L. Guan Y. Yam L. Lim W. (2003). Coronavirus as a possible cause of severe acute respiratory syndrome. LANCET 361 (9366), 1319–1325. 10.1016/s0140-6736(03)13077-2 12711465 PMC7112372

[B50] Qin G. Zhao C. Liu Y. Zhang C. Yang G. Yang J. (2022). RNA G-quadruplex formed in SARS-CoV-2 used for COVID-19 treatment in animal models. Cell Discov. 8 (1), 86. 10.1038/s41421-022-00450-x 36068208 PMC9447362

[B51] Ruggiero E. Richter S. N. (2018). G-Quadruplexes and G-quadruplex ligands: Targets and tools in antiviral therapy. Nucleic Acids Res. 46 (7), 3270–3283. 10.1093/nar/gky187 29554280 PMC5909458

[B52] Schneider T. D. Stephens R. M. (1990). Sequence logos: A new way to display consensus sequences. Nucleic Acids Res. 18 (20), 6097–6100. 10.1093/nar/18.20.6097 2172928 PMC332411

[B53] Schoeman D. Fielding B. C. (2020). Is there a link between the pathogenic human coronavirus envelope protein and immunopathology? A review of the literature. Front. Microbiol. 11, 2086. 10.3389/fmicb.2020.02086 33013759 PMC7496634

[B54] Shen W. Gorelick R. J. Bambara R. A. (2011). HIV-1 nucleocapsid protein increases strand transfer recombination by promoting dimeric G-quartet formation. J. Biol. Chem. 286 (34), 29838–29847. 10.1074/jbc.M111.262352 21737842 PMC3191025

[B55] Sisk J. M. Frieman M. B. Machamer C. E. (2018). Coronavirus S protein-induced fusion is blocked prior to hemifusion by Abl kinase inhibitors. J. Gen. Virol. 99 (5), 619–630. 10.1099/jgv.0.001047 29557770 PMC6537626

[B56] Taylor P. C. Adams A. C. Hufford M. M. de la Torre I. Winthrop K. Gottlieb R. L. (2021). Neutralizing monoclonal antibodies for treatment of COVID-19. Nat. Rev. Immunol. 21 (6), 382–393. 10.1038/s41577-021-00542-x 33875867 PMC8054133

[B57] Thiel V. Ivanov K. A. Putics A. Hertzig T. Schelle B. Bayer S. (2003). Mechanisms and enzymes involved in SARS coronavirus genome expression. J. Gen. Virol. 84 (9), 2305–2315. 10.1099/vir.0.19424-0 12917450

[B58] Villar-Guerra R. F. Trent J. O. Chaires J. B. (2018) ‘G-quadruplex secondary structure obtained from circular dichroism spectroscopy’ 10.1002/anie.201709184 PMC5920796 29076232

[B59] Walls A. C. Park Y. J. Tortorici M. A. Wall A. McGuire A. T. Veesler D. (2020). Structure, function, and antigenicity of the SARS-CoV-2 spike glycoprotein. Cell 181 (2), 281–292.e6. e6. 10.1016/j.cell.2020.02.058 32155444 PMC7102599

[B60] Wang M. Cao R. Zhang L. Yang X. Liu J. Xu M. (2020). Remdesivir and chloroquine effectively inhibit the recently emerged novel coronavirus (2019-nCoV) *in vitro* . Cell Res. 30 (3), 269–271. 10.1038/s41422-020-0282-0 32020029 PMC7054408

[B61] Wang S.-R. Min Y. Q. Wang J. Q. Liu C. X. Fu B. S. Wu F. (2016). A highly conserved G-rich consensus sequence in hepatitis C virus core gene represents a new anti–hepatitis C target. Sci. Adv. 2 (4), e1501535. 10.1126/sciadv.1501535 27051880 PMC4820367

[B62] Wang Y. Han G. Jiang X. Yuwen T. Xue Y. (2021). Chemical shift prediction of RNA imino groups: Application toward characterizing RNA excited states. Nat. Commun. 12 (1), 1595. 10.1038/s41467-021-21840-x 33707433 PMC7952389

[B63] Yu C.-H. Teulade-Fichou M.-P. Olsthoorn R. C. L. (2014). Stimulation of ribosomal frameshifting by RNA G-quadruplex structures. Nucleic Acids Res. 42 (3), 1887–1892. 10.1093/nar/gkt1022 24178029 PMC3919603

[B64] Zaki A. M. van Boheemen S. Bestebroer T. M. Osterhaus A. D. Fouchier R. A. (2012). Isolation of a novel coronavirus from a man with pneumonia in Saudi Arabia. N. Engl. J. Med. Overseas. Ed. 367 (19), 1814–1820. 10.1056/NEJMoa1211721 23075143

[B65] Zhao C. Qin G. Niu J. Wang Z. Wang C. Ren J. (2021). Targeting RNA G‐quadruplex in SARS‐CoV‐2: A promising therapeutic target for COVID‐19? Angew. Chem. Int. Ed. 60 (1), 432–438. 10.1002/anie.202011419 32939952

[B66] Zhou W. Wang W. (2021). Fast-spreading SARS-CoV-2 variants: Challenges to and new design strategies of COVID-19 vaccines. Signal Transduct. Target. Ther. 6 (1), 226. 10.1038/s41392-021-00644-x 34108440 PMC8187888

[B67] Zhu F.-C. Li Y. H. Guan X. H. Hou L. H. Wang W. J. Li J. X. (2020). Safety, tolerability, and immunogenicity of a recombinant adenovirus type-5 vectored COVID-19 vaccine: A dose-escalation, open-label, non-randomised, first-in-human trial. Lancet 395 (10240), 1845–1854. 10.1016/S0140-6736(20)31208-3 32450106 PMC7255193

[B68] Zhu N. Zhang D. Wang W. Li X. Yang B. Song J. (2020). A novel coronavirus from patients with pneumonia in China, 2019. N. Engl. J. Med. Overseas. Ed. 382 (8), 727–733. 10.1056/NEJMoa2001017 PMC7092803 31978945

[B69] Ziegler C. G. K. Allon S. J. Nyquist S. K. Mbano I. M. Miao V. N. Tzouanas C. N. (2020). SARS-CoV-2 receptor ACE2 is an interferon-stimulated gene in human airway epithelial cells and is detected in specific cell subsets across tissues. Cell 181 (5), 1016–1035.e19. e19. 10.1016/j.cell.2020.04.035 32413319 PMC7252096

