# Supplementary material for: RNA G-quadruplex forming regions from SARS-2, SARS-1 and MERS coronoviruses
Source: Front Chem. 2022 Nov 21;10:1014663. doi: 10.3389/fchem.2022.1014663 (PMC9719988; doi:10.3389/fchem.2022.1014663)
Supplement: Supplementary file 1 [file DataSheet1.PDF]

Table S1: Putative G4 Forming Sequences detected in SARS-CoV-2 by G4-hunter algorithm**

Start*	End*	Sequence	Length	Average Score
**786**	806	GCGTGAGCTTAACGGAGGGG	20	1.05
**2452**	2474	CTCATGCCTCTAAAAGCCCCAA	22	-0.95
**4254**	4274	GGGTCAGGGTTTAAATGGTT	20	1.05
**4503**	4523	GGGTGTGGTTGATTATGGTG	20	1.0
**13108**	13129	GATTATCTAGCTAGTGGGGGA	21	1.0
**14954**	14974	ATTTAATAAATGGGGTAAGG	20	1.0
**15921**	15943	TACCTTCCTTACCCAGATCCAT	22	-0.91
**16232**	16255	ACAGGCTGTTGGGGCTTGTGTTC	23	0.83
**18298**	18320	TTGGCTTCGATGTCGAGGGGTG	22	0.95
**21618**	21654	AACCAGAACTCAATTACCCCCTGCATACACTAATTC	36	-0.78
**23499**	23519	TGCAGGCTGTTTAATAGGGG	20	1.0
**25691**	25711	CCTTGAAGCCCCTTTTCTCT	20	-1.0
**28356**	28380	GAATGGAGAACGCAGTGGGGCGCG	24	0.92
**28396**	28416	CCCCAAGGTTTACCCAATAA	20	-1.05
**29121**	29142	AGGAAATTTTGGGGACCAGGA	21	0.95
**29183**	29203	TTGCACAATTTGCCCCCAGC	20	-1.0

Table S2: Putative G4 Forming Sequences detected in SARS-CoV by G4-Hunter algorithm**

Start*	End*	Sequence	Length	Average Score
**349**	371	GTGGCTTCGGGGACTCTGTGGA	22	1.0
**444**	468	CTGCCCCAGCTTGAACAGCCCTAT	24	-1.0
**599**	619	CGAAACCCCAATTGCATACC	20	-1.0
**601**	624	AAACCCCAATTGCATACCGCAAT	23	-0.87
**1137**	1166	AAGACTGAGGGTTTCATGGGGCGTATACG	29	0.86
**2712**	2732	GGGGGTGCACCAATTAAAGG	20	1.0
**10261**	10281	CTTCTAACCCTAAGACACCC	20	-1.0
**12721**	12742	AGGGAGGTAGGTTTGTGCTGG	21	1.05
**14884**	14904	ATTTAATAAATGGGGTAAGG	20	1.0
**16304**	16325	CCCTATGTTTGCAATGCCCCA	21	-1.1
**17368**	17406	TCCTGCTCAATTACCAGCCCCCCGCACATTGCTGACTA	38	-0.87
**18700**	18726	TCAGCAGTGGGGCTTTACGGGTAACC	26	0.73
**21193**	21213	GGAAGCATTTTTAATTGGGG	20	1.0
**21589**	21623	TACTTCATCTATGAGGGGGGTTTACTATCCTGAT	34	0.53
**22880**	22913	GGCAAACCTTGCACCCCACCTGCTCTTAATTGT	33	-0.64
**23215**	23245	TTGCGCTTTTGGGGGTGTAAGTGTAATTAC	30	0.73
**24575**	24597	TACCACCTTATGTCCTTCCCAC	22	-0.95
**24585**	24612	TGTCCTTCCCACAAGCAGCCCCGCATG	27	-1.0
**26611**	26637	TTAATTGGGTGACTGGCGGGATTGCG	26	0.85
**28133**	28154	ACCCCAATCAAACCAACGTAG	21	-0.95
**28204**	28231	AGAATGGAGGACGCAATGGGGCAAGGC	27	0.96
**28229**	28251	GCCAAAACAGCGCCGACCCCAA	22	-1.0

** Sequences selected for *in vitro* biophysical analysis appear in red

* Start/End is the position of the sequence in the alignment
a window of 20 nucleotides was chosen for the algorithm

Table S3: Putative G4 Forming Sequences detected in MERS-CoV by G4-hunter algorithm**.

Start	End	Sequence	Length	Average Score
**20**	45	CTATCTCACTTCCCCTCGTTCTCTT	25	-0.88
**252**	274	CGGTGCGTGGCAATTCGGGGCA	22	0.95
**733**	762	TTATCACTACACCCCATTCCACTATGAGC	29	-0.79
**1158**	1180	TAACGCTCACCCCACGCAACAA	22	-0.91
**1337**	1359	GGGTTTGCCTGTGGATGTGGGG	22	1.27
**2345**	2365	GGGTTTGTGGTGGTCAATGG	20	1.05
**3147**	3167	TGATCTTCTCTCTTCACCCC	20	-1.0
**4939**	4959	CCCCGTTGATCCTACTTTCT	20	-1.0
**4992**	5014	ATAAGTGGAAGATGGTTGTGTG	22	0.95
**6126**	6146	CCCCCATTGAACTCGAAAAT	20	-1.0
**7548**	7570	GTGACACTGCAGGTGTGGGGAA	22	0.95
**8987**	9007	ATGTTTAGGGATGCAGAGGG	20	1.0
**11195**	11215	CCCACTACTCCCATTTCGTC	20	-1.05
**11240**	11264	CTTGCCCCCACTAATGCTTATATG	24	-0.83
**11412**	11437	AAGCCTCAAGCCCCATTGCCTATCT	25	-0.92
**12507**	12535	ACTACGCTGGGGCTTTGTGGGACATTAC	28	0.75
**13326**	13346	CAAATACCCCCTGTAATGTC	20	-1.0
**13403**	13423	CCCCAATCTAAAGATTCCAA	20	-1.0
**15608**	15638	GGAGCACTAGCCCAGACCCCAAATTTGTTG	30	-0.6
**15892**	15914	TTCCTTCCTTATCCAGACCCTT	22	-0.91
**18384**	18406	GTGGGGTAACATGTTAACGGGC	22	1.09
**18461**	18485	ATAAGGGGGCTGCGTGGCCTATTG	24	0.88
**18733**	18758	CAACAGTGGGGTTATGTAGGCAATC	25	0.72
**19414**	19434	ACAGCTTGTAATTTAGGGGG	20	1.0
**20164**	20190	AGTGACTTCCTACCCCTTTCTGACAT	26	-0.77
**23564**	23589	TATGGCCCCCTTCAGACACCTGTTG	25	-0.76
**24934**	24955	CCCTACTAATTGTATAGCCCC	21	-1.14
**25032**	25061	CACCTGAGCCCATTACCTCCCTTAATACT	29	-0.9
**25096**	25125	TTCTACTAACCTCCCTCCTCCTCTTCTCG	29	-0.86
**25920**	25942	TACATCCCTAAACCCACAGCTA	22	-0.91
**26863**	26884	AAACCCGTCCAGCTAGTCCCA	21	-0.95
**27475**	27496	CCTCTTCACATAATCGCCCCG	21	-1.05
**27799**	27829	GATAGTAAACCCCCTCTACCACCTGACGAG	30	-0.83
**28569**	28589	CATCCCCTGCTGCACCTCGT	20	-1.05
**28721**	28742	GTCCCTCTTACCTTTCCACCT	21	-1.0
**28757**	28778	CTTAATGCCAATTCTACCCCT	21	-1.0
**28789**	28810	TGGGTATTGGCGGAGACAGGA	21	0.95
**29045**	29073	CACATTGAGGGGACTGGAGGCAATAGTC	28	0.75
**29433**	29454	ACCCACGTTGGCCCCAAATTG	21	-0.95
**29848**	29874	GGCAACCCCATCTCACCATCGCTTGT	26	-0.73

** Sequences selected for *in vitro* biophysical analysis appear in red

* Start/End is the position of the sequence in the alignment
a window of 20 nucleotides was chosen for the algorithm

Figure S1: Summary of the G4-hunter results for SARS-CoV-2

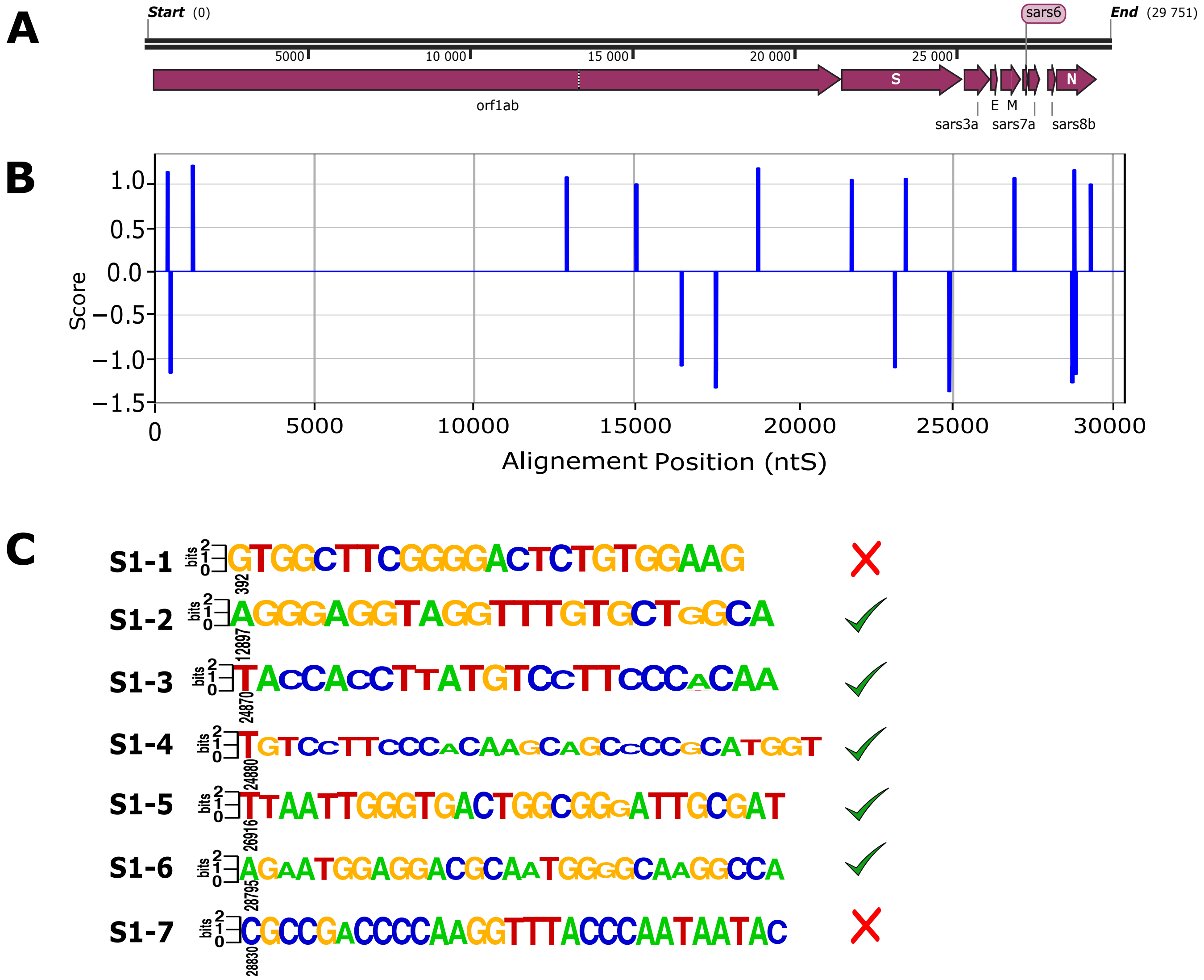

(A) SARS-COV-2 genome. (B) Graphical representation of the G4-hunter average score of 2500 aligned sequences. The positive bars in the graph represent the sequences detected having a score ≧+1 (located in the (+) strand of the viral genome) and the negative bars represent the sequences detected having a score ≦1 (located in the (–) strand of the viral genome). (C) Logo representation of the putative G4 forming sequences selected for biophysical analysis. This representation using weblogo software depicts nucleotide conservation in the alignment (2500 sequences). The green check shows that the sequence is able to form a G-quadruplex *in vitro* as demonstrated by the biophysical analysis. The red cross shows that the sequence does not form a G-quadruplex *in vitro* as demonstrated by the biophysical analysis.

Figure S2: Summary of the G4hunter results for SARS-CoV.

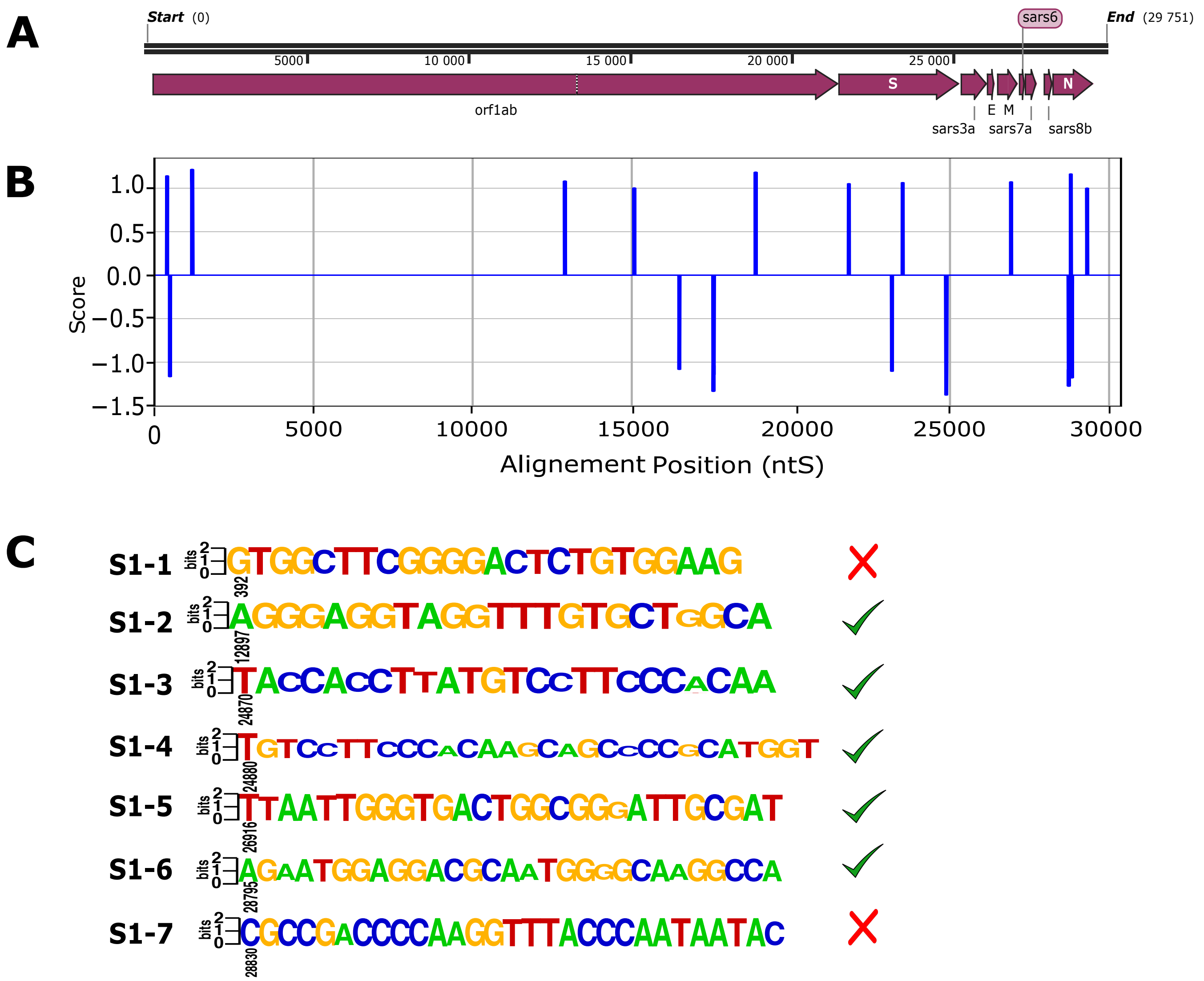

(A) SARS-COV genome. (B) Graphical representation of the G4-hunter average score of 308 aligned sequences. The positive bars in the graph represent the sequences detected having a score ≧+1 (located in the (+) strand of the viral genome) and the negative bars represent the sequences detected having a score ≦1 (located in the (–) strand of the viral genome). (C) Logo representation of the putative G4 forming sequences selected for biophysical analysis. This representation using weblogo software depicts nucleotide conservation in the alignment (308 sequences). The green check shows that the sequence is able to form a G-quadruplex *in vitro* as demonstrated by the biophysical analysis. The red cross shows that the sequence does not form a G-quadruplex *in vitro* as demonstrated by the biophysical analysis.

Figure S3: Summary of the G4hunter results for MERS-CV

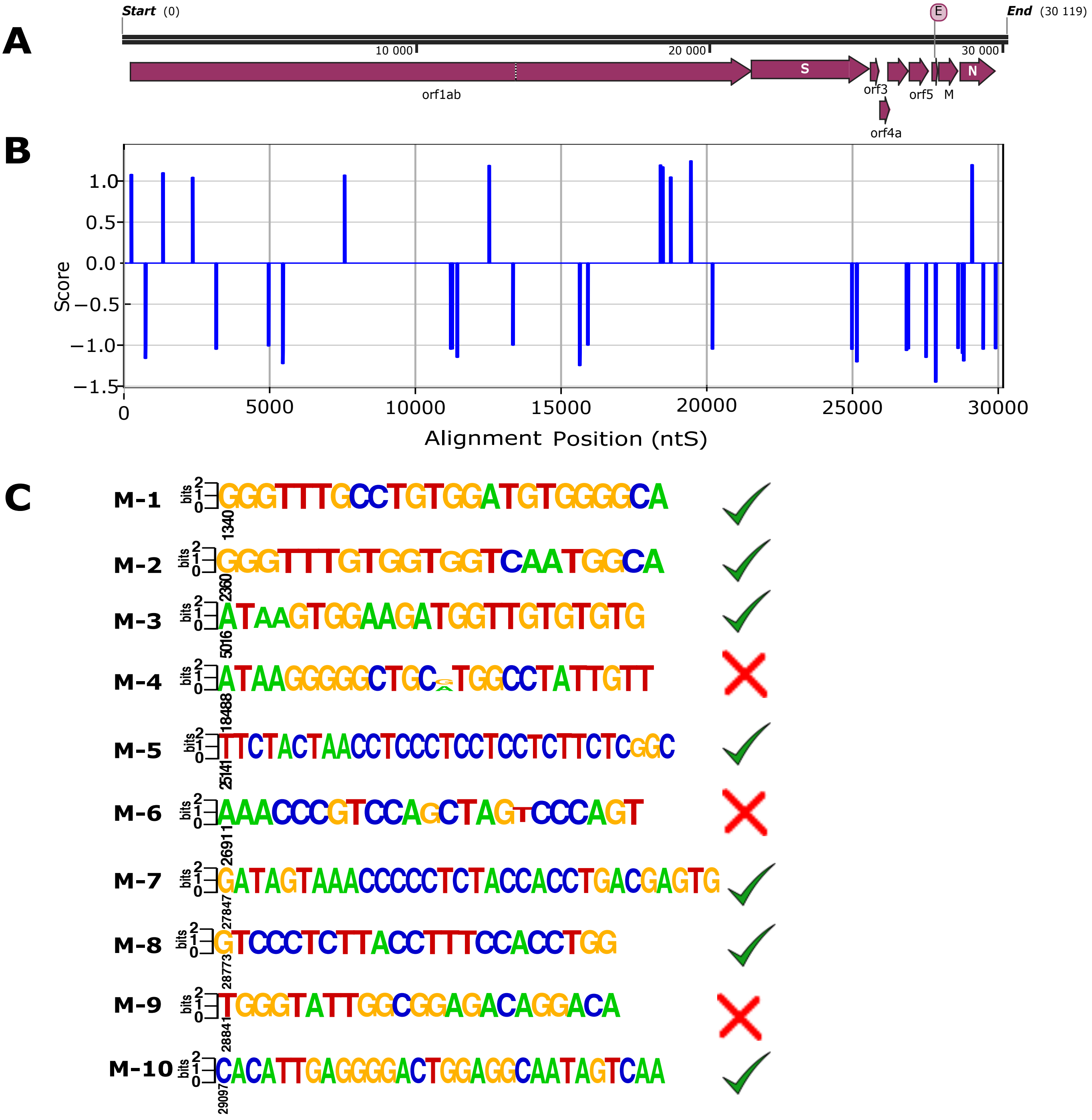

(A) SARS-COV genome. (B) Graphical representation of the G4-hunter average score of 459 aligned sequences. The positive bars in the graph represent the sequences detected having a score ≧+1 (located in the (+) strand of the viral genome) and the negative bars represent the sequences detected having a score ≦1 (located in the (–) strand of the viral genome). (C) Logo representation of the putative G4 forming sequences selected for biophysical analysis. This representation using weblogo software depicts nucleotide conservation in the alignment (459 sequences). The green check shows that the sequence is able to form a G-quadruplex *in vitro* as demonstrated by the biophysical analysis. The red cross shows that the sequence does not form a G-quadruplex *in vitro* as demonstrated by the biophysical analysis.

Figure S4A

Name	Sequence (5'-3')[a]	Length (nt)	RNA Strand +/-	Position (nt)	CD[b]	TDS[c] +/-	T_m (°C)[d]	NMR[e] (YES/NO)	G4?[f] YES/NO
S2-1	GCGUGAGCUUAACGGAGGGGC	21	(+)	786	folded	(+)	40 (295 nm)	yes	yes

a. Sequence retrieved from the reference isolate for each virus
b. CD: circular dichroism. Folded/unfolded depending on the intensity of the CD spectra. nd: not determined
c. TDS: Thermal differential spectrum. (+) indicates the presence of a peak at 295 nm. (-) indicate the absence of the peak.
d. T_m: Thermal melting temperature (the standard error is of ±1°C). Two Tm values are indicated when the melting process presents an hysteresis.
e. NMR: Yes indicates the presence of imino proton resonances. No indicates the absence of imino protons resonances
f. G4? Yes or No indicate that the sequence forms or not a G4 according to the data of CD, TDS, Tm. Hp means that a RNA hairpin structure is predicted by VIENNA software. G4<->hp : we speculate a G4/Hairpin equilibrium

Figure S4B: Biophysical *In vitro* characterization

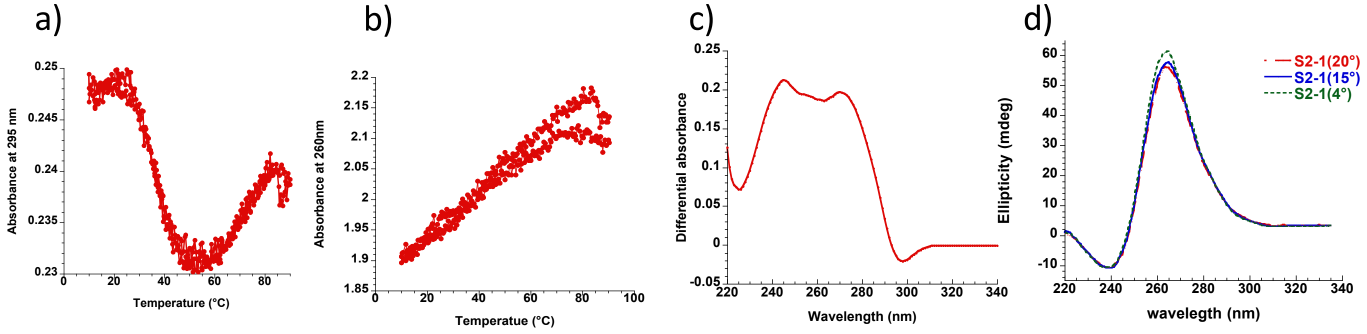

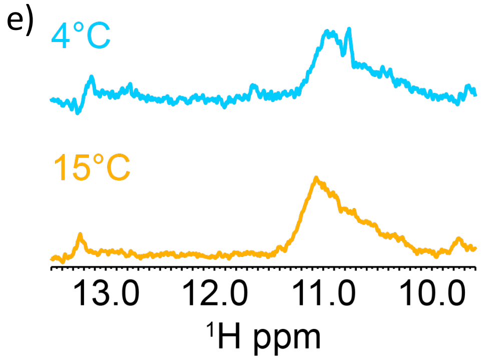

Analysis: T_m : At 295 nm the heating and cooling profiles are superimposed, no hysteresis is observed. Thus, the folding and unfolding processes are kinetically fast suggesting the formation of a mono-molecular G4 structure. At 260 nm, no cooperative structural transition is observed. **TDS**: The hypo-chromicity around 295 nm suggests the formation of a G4 fold. **CD** : The intense positive peak at 267 nm suggests the oligonucleotide is well folded. **NMR**: At 4°C and 15°C, imino proton peaks were detected in the [10 – 12] ppm region suggesting the formation of G-tetrads. One peak is present around 13 ppm suggesting the formation of a AU base-pair.

In vitro characterization of the selected candidates. Experiments were performed at around 4 µM (CD, TDS, UV-melting) dissolved in 10 mM lithium cacodylate pH 7 and 120 mM KCl, or 100 µM RNA strand concentration (NMR) dissolved in 20 mM Potassium phosphate pH7 and 120 mM KCl: **a)** Thermal melting transition measured at 295 nm . **b)** Thermal melting profiles measured at 260 nm **c)** Thermal differential Spectra (TDS). d) Circular Dichroism (CD) spectra recorded at 4°C, 15°C, 20°C. **e)** 1D 1H-NMR spectrum of the imino proton region recorded at 15°C and 4°C.

Figure S5A

Name	Sequence (5'-3')[a]	Length (nt)	RNA Strand +/-	Position (nt)	CD[b]	TDS[c] +/-	T_m (°C)[d]	NMR (YES/NO)	G4?[f] YES/NO
S2-2	UGGAGGAGGUGUUGCAGGA	19	(+)	3466	folded	(+)	40/44 (295 nm)	yes	yes

a. Sequence retrieved from the reference isolate for each virus

b. CD: circular dichroism. Folded/unfolded depending on the intensity of the CD spectra. nd: not determined

c.TDS: Thermal differential spectrum. (+) indicates the presence of a peak at 295 nm. (-) indicate the absence of the peak.

d.T_m: Thermal melting temperature (the standard error is of ±1°C). Two Tm values are indicated when the melting process presents an hysteresis.

e. NMR: Yes indicates the presence of imino proton resonances. No indicates the absence of imino protons resonances

f. G4? Yes or No indicate that the sequence forms or not a G4 according to the data of CD, TDS, Tm. Hp means that a RNA hairpin structure is predicted by VIENNA software. G4<->hp : we speculate a G4/Hairpin equilibrium

Figure S5B: Biophysical *In vitro* characterization

a)

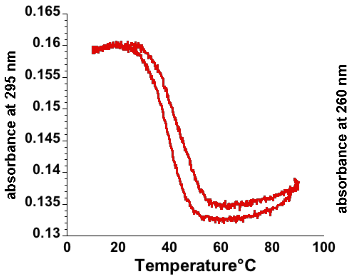

b)

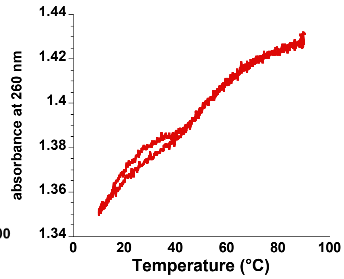

c)

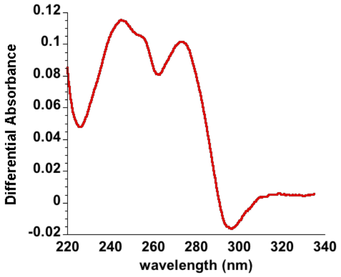

d)

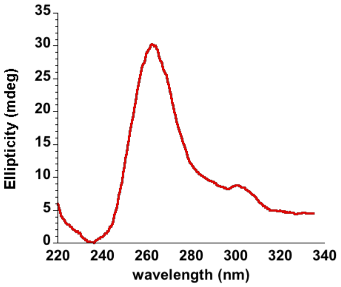

e)

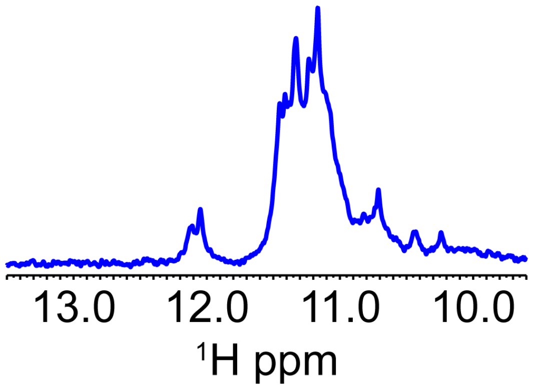

Analysis: T_m: At 295 nm, the structure is stable and the melting process present a hysteresis of 4°C. At 260 nm, the process also present a hysteresis at low temperature. **TDS** : The hypochromicity at 295 nm suggests the formation of a G4 fold. **CD** : The positive peak at 267 nm suggests that the oligonucleotide is well folded. NMR: the presence of a set of well resolved peak in the [10 – 12] ppm region confirms the formation of G4s with a limited conformational polymorphism. Peaks in the [12 – 13] ppm region suggests the formation of additional CG base pairs

In vitro characterization of the selected candidates. Experiments were performed at around 4 µM (CD, TDS, UV-melting) dissolved in 10 mM lithium cacodylate pH 7 and 120 mM KCl, or 100 µM RNA strand concentration (NMR) dissolved in 20 mM Potassium phosphate pH7 and 120 mM KCl: **a)** Thermal melting transition measured at 295 nm . **b)** Thermal melting profiles measured at 260 nm **c)** Thermal differential Spectra (TDS). **d)** Circular Dichroism (CD) spectra . **e)** 1D 1H-NMR spectrum of the imino proton region recorded at 15°C.

Figure S6A

Name	Sequence (5'-3')[a]	Length (nt)	RNA Strand +/-	Position (nt)	CD[b]	TDS[c] +/-	T_m (°C)[d]	NMR (YES/NO)	G4?[f] YES/NO
S2-3	GGGUCAGGGUUUAAAUGGU	19	(+)	4254	folded	(+)	35/55 (295 nm)	yes	yes

a. Sequence retrieved from the reference isolate for each virus
b. CD: circular dichroism. Folded/unfolded depending on the intensity of the CD spectra. nd: not determined
c.TDS: Thermal differential spectrum. (+) indicates the presence of a peak at 295 nm. (-) indicate the absence of the peak.
d.T_m: Thermal melting temperature (the standard error is of ±1°C). Two Tm values are indicated when the melting process presents an hysteresis.
e. NMR: Yes indicates the presence of imino proton resonances. No indicates the absence of imino protons resonances
f. G4? Yes or No indicate that the sequence forms or not a G4 according to the data of CD, TDS, Tm. Hp means that a RNA hairpin structure is predicted by VIENNA software. G4<->hp : we speculate a G4/Hairpin equilibrium

Figure S6B: Biophysical *In vitro* characterization

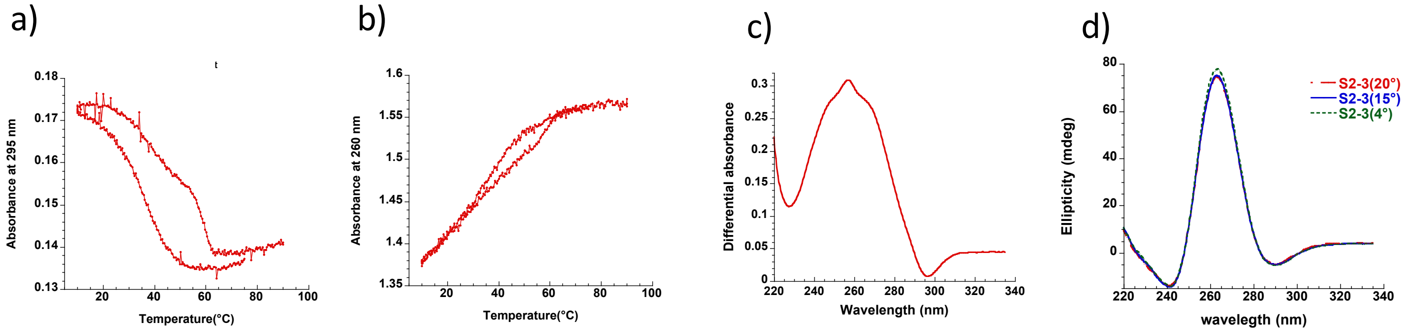

e)

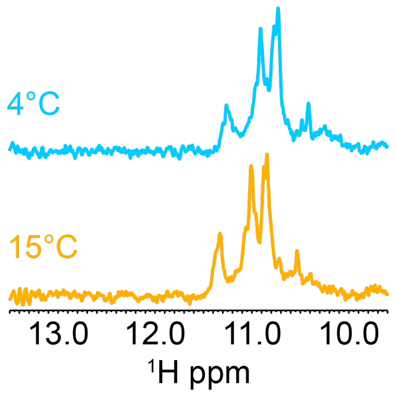

Analysis: T_m : : At 295 nm, the hypochromic melting process is not reversible and present an hysteresis of 20°C. Thus, the folding and unfolding processes are kinetically slow suggesting the formation of a multi-molecular G4 structure. Similarly at 260 nm, the process is also not reversible with the same hysteresis. **TDS**: The hypochromicity around 295 nm suggests that the oligonucleotide adopts of a G4 fold. **CD** : The intense positive peak at 267 nm suggests that the RNA oligonucleotide is well folded. **NMR:** At 4°C and 15°C, imino proton peaks were detected in the [10 – 12] ppm region suggesting the formation of G-tetrads.

In vitro characterization of the selected candidates. Experiments were performed at around 4 µM (CD, TDS, UV-melting) dissolved in 10 mM lithium cacodylate pH 7 and 120 mM KCl, or 100 µM RNA strand concentration (NMR) dissolved in 20 mM Potassium phosphate pH7 and 120 mM KCl: **a)** Thermal melting transition measured at 295 nm . **b)** Thermal melting profiles measured at 260 nm **. c) Thermal** differential Spectra (TDS). **d)** Circular Dichroism (CD) spectra recorded at 4°C, 15°C, 20°C. **e)** 1D 1H-NMR spectrum of the imino proton region recorded at 15°C and 4°C.

Figure S7A

Name	Sequence (5'-3')[a]	Length (nt)	RNA Strand +/-	Position (nt)	CD[b]	TDS[c] +/-	T_m (°C)[d]	NMR (YES/NO)	G4?[f] YES/NO
S2-4	AGGGUGUGGUUGAUUAUGGUG	21	(+)	4503	folded	(+)	28/33 (295 nm)	yes	yes

a. Sequence retrieved from the reference isolate for each virus
b. CD: circular dichroism. Folded/unfolded depending on the intensity of the CD spectra. nd: not determined
c.TDS: Thermal differential spectrum. (+) indicates the presence of a peak at 295 nm. (-) indicate the absence of the peak.
d.T_m: Thermal melting temperature (the standard error is of ±1°C). Two Tm values are indicated when the melting process presents an hysteresis.
e. NMR: Yes indicates the presence of imino proton resonances. No indicates the absence of imino protons resonances
f. G4? Yes or No indicate that the sequence forms or not a G4 according to the data of CD, TDS, Tm. Hp means that a RNA hairpin structure is predicted by VIENNA software. G4<->hp : we speculate a G4/Hairpin equilibrium

Figure S7B: Biophysical *In vitro* characterization

a)

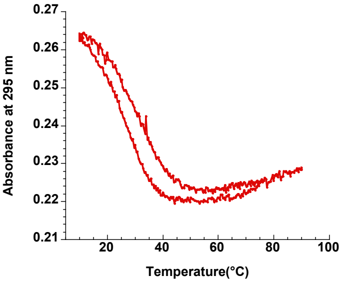

b)

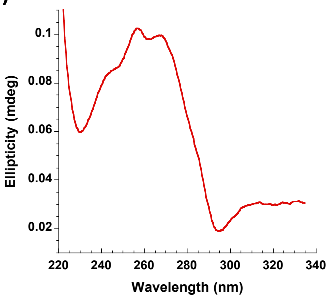

c)

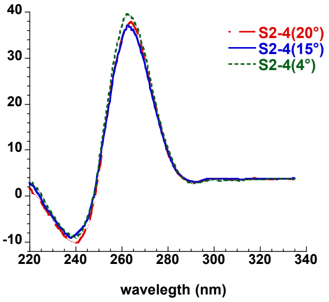

d) NMR

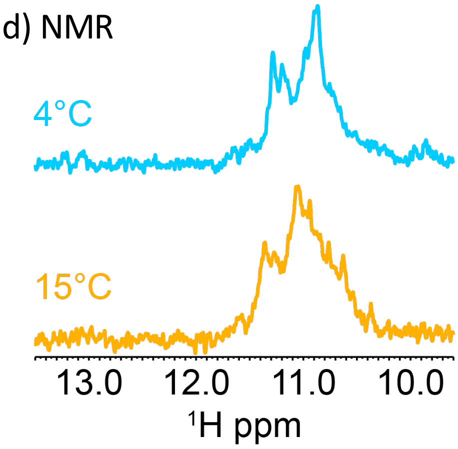

Analysis: T_m : : At 295 nm, the hypochromic melting presents a hysteresis of 5°C suggesting the formation of multi-molecular G4s. **TDS**: The hypochromicity around 295 nm suggests that the oligonucleotide adopts a G4 fold. **CD** : The intense positive peak at 267 nm suggest that the RNA oligonucleotide is well folded. **NMR:** Presence of a very broad NMR signal in the [10 – 12] ppm suggesting the formation of highly polymorphic G4 structures.

Experiments were performed at around 4 µM (CD, TDS, UV-melting) dissolved in 10 mM lithium cacodylate pH 7 and 120 mM KCl, or 100 µM RNA strand concentration (NMR) dissolved in 20 mM Potassium phosphate pH7 and 120 mM KCl: **a)** Thermal melting transition measured at 295 nm . **b) Thermal** differential Spectra (TDS). **c)** Circular Dichroism (CD) spectra recorded at 4°C, 15°C, 20°C. **d)** 1D 1H-NMR spectrum of the imino proton region recorded at 15°C and 4°C.

Figure S8A

Name	Sequence (5’-3’)[a]	Length (nt)	RNA Strand +/-	Position (nt)	CD[b]	TDS[c] +/-	T_m (°C)[d]	NMR (YES/NO)	G4?[f] YES/NO
S2-5	UGGAUCUGGGUAAGGAAGGU	20	(-)	15921	nd	nd	42 (260 nm)	nd	no (hp)

a. Sequence retrieved from the reference isolate for each virus
b. CD: circular dichroism. Folded/unfolded depending on the intensity of the CD spectra. nd: not determined
c.TDS: Thermal differential spectrum. (+) indicates the presence of a peak at 295 nm. (-) indicate the absence of the peak.
d.T_m: Thermal melting temperature (the standard error is of ±1°C). Two Tm values are indicated when the melting process presents an hysteresis.
e. NMR: Yes indicates the presence of imino proton resonances. No indicates the absence of imino protons resonances
f. G4? Yes or No indicate that the sequence forms or not a G4 according to the data of CD, TDS, Tm. Hp means that a RNA hairpin structure is predicted by VIENNA software. G4<->hp : we speculate a G4/Hairpin equilibrium

Figure S8B: Biophysical *In vitro* characterization

a)

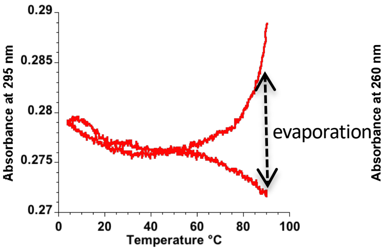

b)

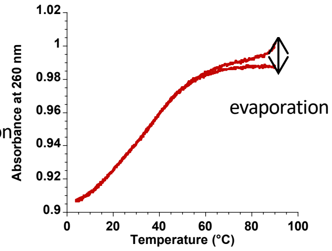

c)

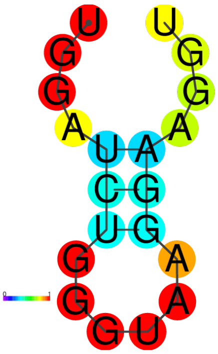

Analysis: T_m: The melting profile does not present any clear transition at 295 nm. At 260 nm, the process is reversible with no hysteresis suggesting the formation of a mono-molecular structure.
TDS : nd. **CD** : nd.
Prediction: a hairpin with 3bp is predicted for this oligonucleotide.

Experiments were performed at around 4 µM (CD, TDS, UV-melting) dissolved in 10 mM lithium cacodylate pH 7 and 120 mM KCl. **a)** Thermal melting transition measured at 295 nm . **b)** Thermal melting profiles measured at 260 nm. **c)** Structure prediction using the RNA fold prediction on the vienna RNA websuite.

Figure S9A

Name	Sequence (5’-3’)[a]	Length (nt)	RNA Strand +/-	Position (nt)	CD[b]	TDS[c] +/-	T_m (°C)[d]	NMR (YES/NO)	G4?[f] YES/NO
S2-6	GGGGUGCAUUUCGCUGAUUUUGGGG	25	(-)	28289	folded	(-)	40 (260 nm)	nd	no (hp)

a. Sequence retrieved from the reference isolate for each virus
b. CD: circular dichroism. Folded/unfolded depending on the intensity of the CD spectra. nd: not determined
c.TDS: Thermal differential spectrum. (+) indicates the presence of a peak at 295 nm. (-) indicate the absence of the peak.
d.T_m: Thermal melting temperature (the standard error is of ±1°C). Two Tm values are indicated when the melting process presents an hysteresis.
e. NMR: Yes indicates the presence of imino proton resonances. No indicates the absence of imino protons resonances
f. G4? Yes or No indicate that the sequence forms or not a G4 according to the data of CD, TDS, Tm. Hp means that a RNA hairpin structure is predicted by VIENNA software. G4<->hp : we speculate a G4/Hairpin equilibrium

Figure S9B: Biophysical *In vitro* characterization

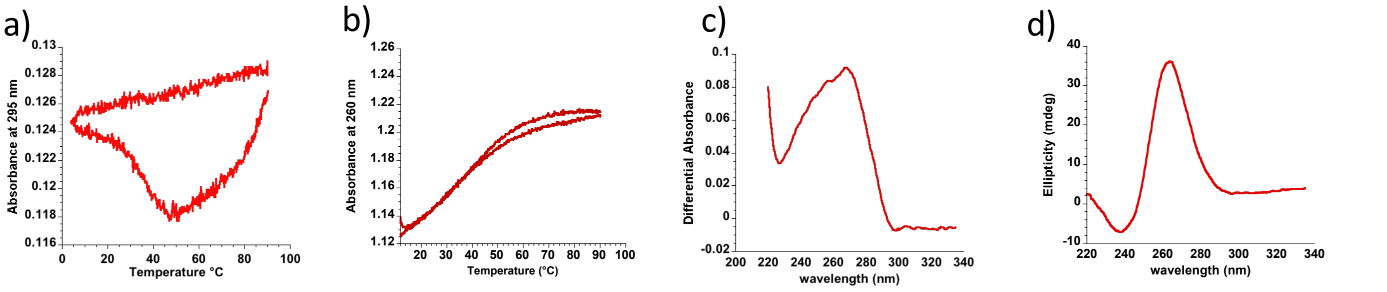

e)

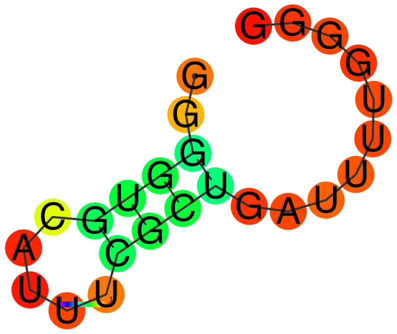

Analysis: **T_m :** At 295 nm, no clear hypochromic transition is detected. At 260 nm, the process presents a slight hysteresis suggesting the formation of multi-molecular and/or mono-molecular structures.
TDS: The absence of hypochromicity around 295 nm suggests the formation of a non-G4 fold. **CD** : The intense positive peak at 267 nm suggests that the RNA is folded and adopts a stable structure. **Prediction: : A** hairpin with 4 bp is predicted for this oligonucleotide.

Experiments were performed at around 4 µM (CD, TDS, UV-melting) dissolved in 10 mM lithium cacodylate pH 7 and 120 mM KCl : **a)** Thermal melting transition measured at 295 nm . **b)** Thermal melting profiles measured at 260 nm **. c) Thermal** differential Spectra (TDS). **d)** Circular Dichroism (CD) spectra **. e) Structure prediction** using the RNA fold prediction on the vienna RNA websuite.

Figure S10A

Name	Sequence (5'-3')[a]	Length (nt)	RNA Strand +/-	Position (nt)	CD[b]	TDS[c] +/-	T_m (°C)[d]	NMR (YES/NO)	G4?[f] YES/NO
S2-7	UGGCUGGCAAUGGCGGU*	17	(+)	28903	FOLDED	(-)	58 (260 nm)	ND	NO (HP)

a. Sequence retrieved from the reference isolate for each virus
b. CD: circular dichroism. Folded/unfolded depending on the intensity of the CD spectra. nd: not determined
c.TDS: Thermal differential spectrum. (+) indicates the presence of a peak at 295 nm. (-) indicate the absence of the peak.
d.T_m: Thermal melting temperature (the standard error is of ±1°C). Two Tm values are indicated when the melting process presents an hysteresis.
e. NMR: Yes indicates the presence of imino proton resonances. No indicates the absence of imino protons resonances
f. G4? Yes or No indicate that the sequence forms or not a G4 according to the data of CD, TDS, Tm. Hp means that a RNA hairpin structure is predicted by VIENNA software. G4<->hp : we speculate a G4/Hairpin equilibrium

Figure S10B: Biophysical *In vitro* characterization

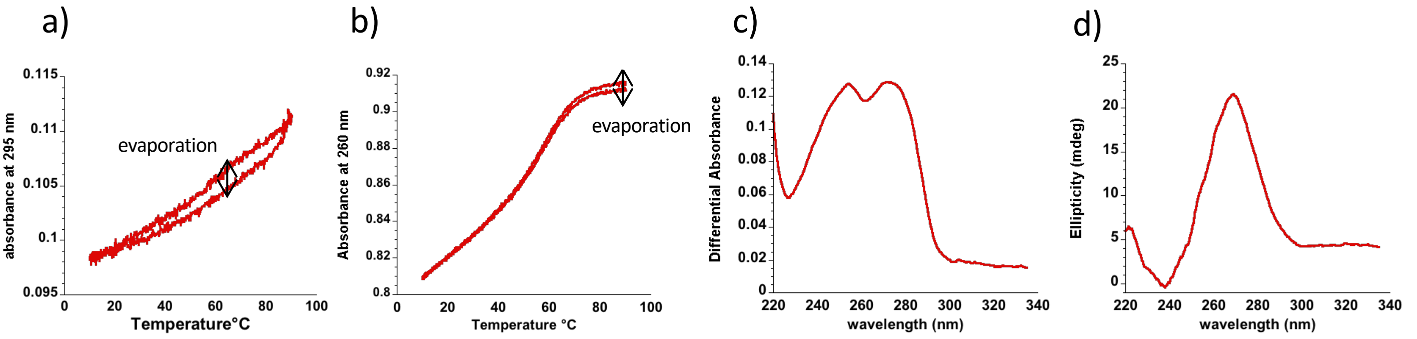

e)

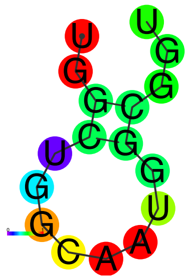

Analysis: T_m: the melting profile at 295 nm does not present a hypochromism and no cooperative structural transition is detected. At 260 nm, the process is reversible with no hysteresis suggesting the formation of a mono-molecular secondary structure. **TDS** : The absence of hypochromicity at 295 nm argue for a non-G4 structure fold. **CD** : The intense positive peak at 268 nm suggest that the structure is folded. **Prediction:** the prediction of this oligonucleotide suggests that it forms a hairpin with 2 GC basepairs but 3 additional GU base pairs are also expected.

Experiments were performed at around 4 µM (CD, TDS, UV-melting) dissolved in 10 mM lithium cacodylate pH 7 and 120 mM KCl : **a)** Thermal melting transition measured at 295 nm . **b)** Thermal melting profiles measured at 260 nm **. c)** Thermal differential Spectra (TDS). **d)** Circular Dichroism (CD) spectra **. e)** Structure prediction using the RNA fold prediction on the vienna RNA websuite.

Figure S11A

Name	Sequence (5'-3')[a]	Length (nt)	RNA Strand +/-	Position (nt)	CD[b]	TDS[c] +/-	T_m (°C)[d]	NMR (YES/NO)	G4?[f] YES/NO
S1-1	GUGGCUUCGGGGACUCUGUGGAAGAGGC	28	(+)	349	folded	(-)	65 (260 nm)	nd	no (hp)

a. Sequence retrieved from the reference isolate for each virus
b. CD: circular dichroism. Folded/unfolded depending on the intensity of the CD spectra. nd: not determined
c.TDS: Thermal differential spectrum. (+) indicates the presence of a peak at 295 nm. (-) indicate the absence of the peak.
d.T_m: Thermal melting temperature (the standard error is of ±1°C). Two Tm values are indicated when the melting process presents an hysteresis.
e. NMR: Yes indicates the presence of imino proton resonances. No indicates the absence of imino protons resonances
f. G4? Yes or No indicate that the sequence forms or not a G4 according to the data of CD, TDS, Tm. Hp means that a RNA hairpin structure is predicted by VIENNA software. G4<->hp : we speculate a G4/Hairpin equilibrium

Figure S11B: Biophysical *In vitro* characterization

a) b) c) d)

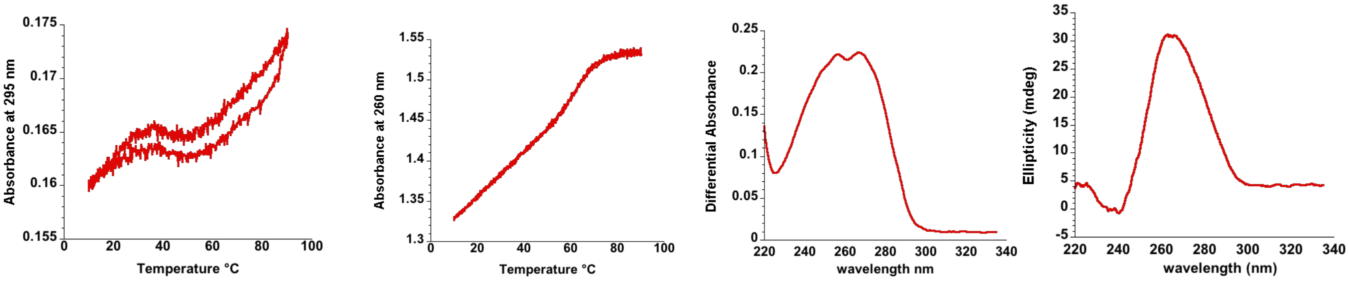

e)

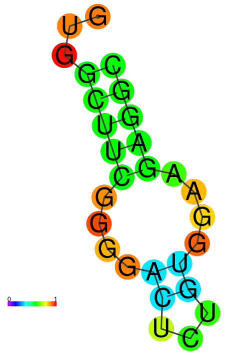

Analysis: T_m: No clear structural transition is detected at 295 nm. At 260 nm, the melting profile presents a cooperative structural transition with no hysteresis suggesting that the structure is mono-molecular. **TDS** : The absence of hypochromicity at 295 nm argue for a non-G4 fold. **CD** : The intense positive peak at 268 nm suggests that the oligonucleotide is well folded. **Prediction:** the prediction of this oligo suggests that it forms a hairpin like structure .

Experiments were performed at around 4 µM (CD, TDS, UV-melting) dissolved in 10 mM lithium cacodylate pH 7 and 120 mM KCl : **a)** Thermal melting transition measured at 295 nm . **b)** Thermal melting profiles measured at 260 nm **. c)** Thermal differential Spectra (TDS). **d)** Circular Dichroism (CD) spectra **. e)** Structure prediction using the RNA fold prediction on the vienna RNA websuite.

Figure S12A

Name	Sequence (5'-3')[a]	Length (nt)	RNA Strand +/-	Position (nt)	CD[b]	TDS[c] +/-	T_m (°C)[d]	NMR (YES/NO)	G4?[f] YES/NO
S1-2	AGGGAGGUAGGUUUGUGCUGG	21	(+)	12721	folded	(+)	41 (295 nm)	yes	yes

a. Sequence retrieved from the reference isolate for each virus
b. CD: circular dichroism. Folded/unfolded depending on the intensity of the CD spectra. nd: not determined
c.TDS: Thermal differential spectrum. (+) indicates the presence of a peak at 295 nm. (-) indicate the absence of the peak.
d.T_m: Thermal melting temperature (the standard error is of ±1°C). Two Tm values are indicated when the melting process presents an hysteresis.
e. NMR: Yes indicates the presence of imino proton resonances. No indicates the absence of imino protons resonances
f. G4? Yes or No indicate that the sequence forms or not a G4 according to the data of CD, TDS, Tm. Hp means that a RNA hairpin structure is predicted by VIENNA software. G4<->hp : we speculate a G4/Hairpin equilibrium

Figure S12B: Biophysical *In vitro* characterization

a) b) c) d)

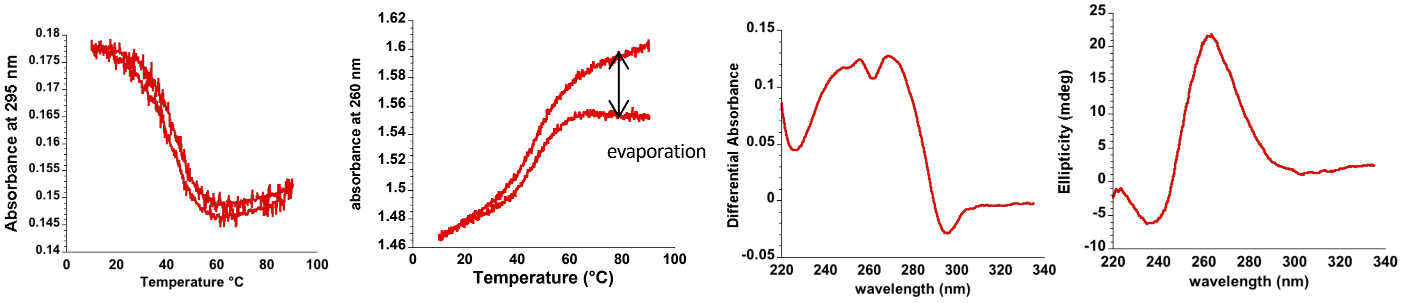

e)

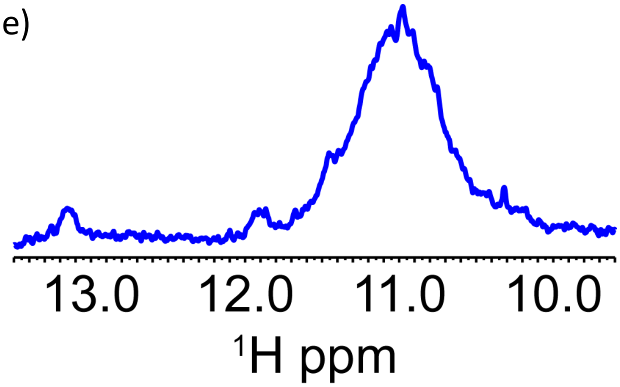

Analysis: T_m: At 295 nm, the structure is stable and the melting process is reversible. At 260 nm, a structural transition is also detected. **TDS** : The hypochromicity at 295 nm suggests the formation of a G4 fold. **CD** : The positive peak at 267 nm suggests that the structure is well folded. **NMR:** Presence of a very broad NMR signal in the [10 - 12] ppm suggesting the formation of highly polymorphic G4 structures.

Experiments were performed at around 4 µM (CD, TDS, UV-melting) dissolved in 10 mM lithium cacodylate pH 7 and 120 mM KCl, or 100 µM RNA strand concentration (NMR) dissolved in 20 mM Potassium phosphate pH7 and 120 mM KCl: **a)** Thermal melting transition measured at 295 nm . **b)** Thermal melting profiles measured at 260 nm **. c)** Thermal differential Spectra (TDS). **d)** Circular Dichroism (CD) spectra. **e)** 1D 1H-NMR spectrum of the imino proton region recorded at 15°C.

Figure S13A

Name	Sequence (5'-3')[a]	Length (nt)	RNA Strand +/-	Position (nt)	CD[b]	TDS[c] +/-	T_m (°C)[d]	NMR (YES/NO)	G4?[f] YES/NO
S1-3	UGUGGGAAGGACAUAAGGUGGUA	23	(-)	24586	FOLDED	(+)	25/50 (295 nm)<br>49 (260 nm)	ND	YES (G4<->HP)

a. Sequence retrieved from the reference isolate for each virus
b. CD: circular dichroism. Folded/unfolded depending on the intensity of the CD spectra. nd: not determined
c.TDS: Thermal differential spectrum. (+) indicates the presence of a peak at 295 nm. (-) indicate the absence of the peak.
d.T_m: Thermal melting temperature (the standard error is of ±1°C). Two Tm values are indicated when the melting process presents an hysteresis.
e. NMR: Yes indicates the presence of imino proton resonances. No indicates the absence of imino protons resonances
f. G4? Yes or No indicate that the sequence forms or not a G4 according to the data of CD, TDS, Tm. Hp means that a RNA hairpin structure is predicted by VIENNA software. G4<->hp : we speculate a G4/Hairpin equilibrium

Figure S13B: Biophysical *In vitro* characterization

a)

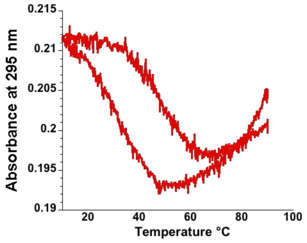

b)

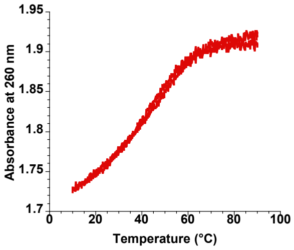

c)

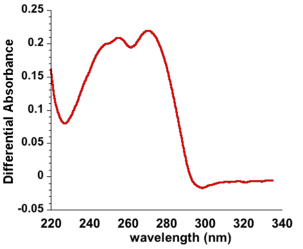

d)

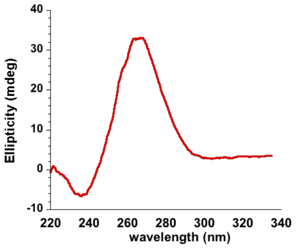

e)

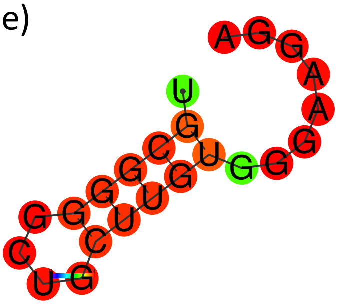

Analysis: T_m : At 295 nm, the hypochromic melting process is not reversible and present an hysteresis of 25°C. Thus, the folding and unfolding processes are kinetically slow suggesting the formation of a multi-molecular G4 structure. At 260 nm, the melting temperature is higher and the process is reversible suggesting that another mono-molecular structure is formed.
TDS: The weak hypochromicity around 295 nm suggests that only a small proportion of the oligonucleotide adopts of a G4 fold. **CD** : The intense positive peak at 267 nm suggest that the RNA oligonucleotide is well folded. **Prediction:** hairpin like structure with 5 base pairs.

Experiments were performed at around 4 µM (CD, TDS, UV-melting) dissolved in 10 mM lithium cacodylate pH 7 and 120 mM KCl : **a)** Thermal melting transition measured at 295 nm . **b)** Thermal melting profiles measured at 260 nm **. c)** Thermal differential Spectra (TDS). **d)** Circular Dichroism (CD) spectra **. e)** Structure prediction using the RNA fold prediction on the vienna RNA websuite.

Figure S14A

Name	Sequence (5'-3')[a]	Length (nt)	RNA Strand +/-	Position (nt)	CD[b]	TDS[c] +/-	T_m (°C)[d]	NMR (YES/NO)	G4?[f] YES/NO
S1-4	UGCGGGGCUGCUUGUGGGAAGGA	23	(-)	24575	folded	(+)	42 (295 nm)	yes	yes

a. Sequence retrieved from the reference isolate for each virus
b. CD: circular dichroism. Folded/unfolded depending on the intensity of the CD spectra. nd: not determined
c.TDS: Thermal differential spectrum. (+) indicates the presence of a peak at 295 nm. (-) indicate the absence of the peak.
d.T_m: Thermal melting temperature (the standard error is of ±1°C). Two Tm values are indicated when the melting process presents an hysteresis.
e. NMR: Yes indicates the presence of imino proton resonances. No indicates the absence of imino protons resonances
f. G4? Yes or No indicate that the sequence forms or not a G4 according to the data of CD, TDS, Tm. Hp means that a RNA hairpin structure is predicted by VIENNA software. G4<->hp : we speculate a G4/Hairpin equilibrium

Figure S14B: Biophysical *In vitro* characterization

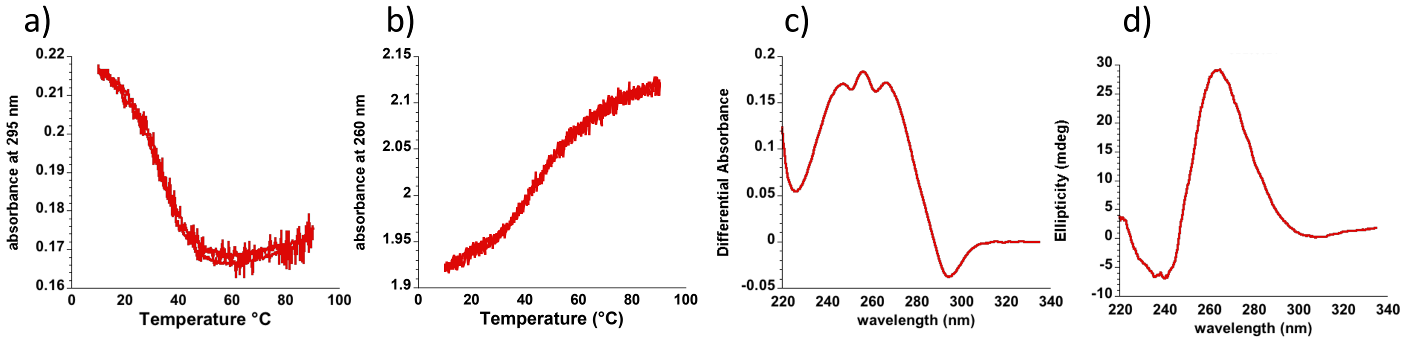

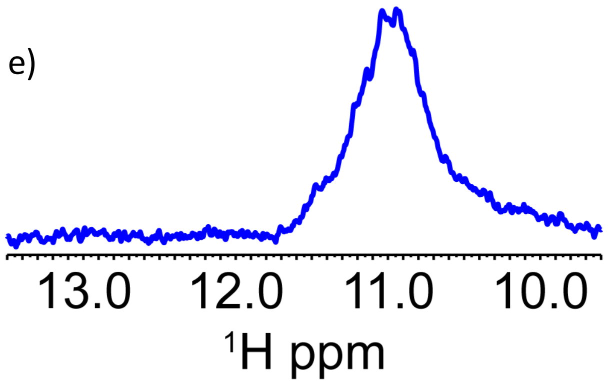

Analysis: T_m : At 295 nm, the structure is stable and the melting process presents a cooperative and reversible transition. At 260 nm, the process is also reversible and presents a cooperative structural transition with the same melting temperature as registered at 295 nm. **TDS** : The hypochromicity at 295 nm suggests the formation of a G4 fold. **CD** : The intense positive peak at 267 nm suggest that the oligonucleotide is folded. **NMR:** Presence of a very broad NMR signal in the [10 – 12] ppm suggesting the formation of highly polymorphic G4 structures.

Experiments were performed at around 4 µM (CD, TDS, UV-melting) dissolved in 10 mM lithium cacodylate pH 7 and 120 mM KCl, or 100 µM RNA strand concentration (NMR) dissolved in 20 mM Potassium phosphate pH7 and 120 mM KCl: **a)** Thermal melting transition measured at 295 nm . b) Thermal melting profiles measured at 260 nm **. c) Thermal** differential Spectra (TDS). **d**) Circular Dichroism (CD) spectra. **e)** 1D 1H-NMR spectrum of the imino proton region recorded at 15°C.

Figure S15A

Name	Sequence (5'-3')[a]	Length (nt)	RNA Strand +/-	Position (nt)	CD[b]	TDS[c] +/-	T_m (°C)[d]	NMR (YES/NO)	G4?[f] YES/NO
S1-5	UGGGUGACUGGCGGGA	16	(+)	26611	folded	(+)	45/55 (295 nm)	yes	yes

a. Sequence retrieved from the reference isolate for each virus
b. CD: circular dichroism. Folded/unfolded depending on the intensity of the CD spectra. nd: not determined
c.TDS: Thermal differential spectrum. (+) indicates the presence of a peak at 295 nm. (-) indicate the absence of the peak.
d.T_m: Thermal melting temperature (the standard error is of ±1°C). Two Tm values are indicated when the melting process presents an hysteresis.
e. NMR: Yes indicates the presence of imino proton resonances. No indicates the absence of imino protons resonances
f. G4? Yes or No indicate that the sequence forms or not a G4 according to the data of CD, TDS, Tm. Hp means that a RNA hairpin structure is predicted by VIENNA software. G4<->hp : we speculate a G4/Hairpin equilibrium

Figure S15B: Biophysical *In vitro* characterization

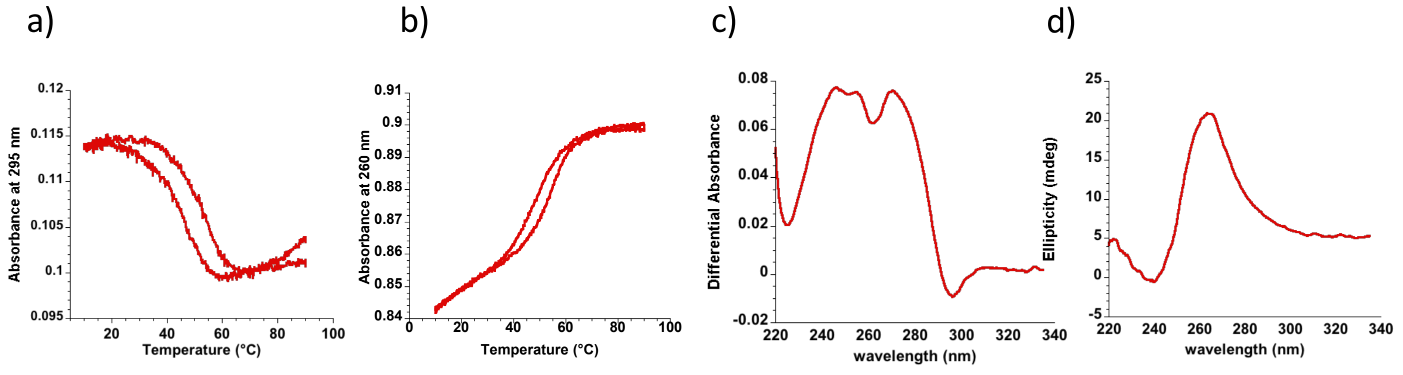

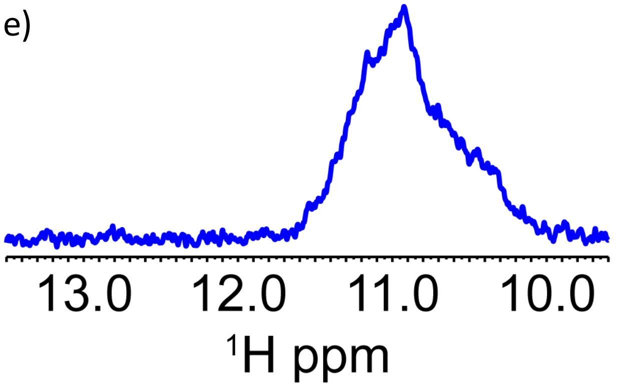

Analysis: T_m: At 295 nm and 260 nm, the process is not reversible. The heating and cooling profiles have 2 different melting temperatures. This hysteresis of 10°C shows that the folding and unfolding processes are kinetically slow suggesting the presence of a majority of multi-molecular structures.
TDS : The hypochromicity at 295 nm suggests the formation of a G4 fold. **CD** : The intense positive peak at 267 nm suggest that the structure is a well folded. **NMR:** Presence of a very broad NMR signal in the [10 – 12] ppm suggesting the formation of highly polymorphic G4 structures.

Experiments were performed at around 4 µM (CD, TDS, UV-melting) dissolved in 10 mM lithium cacodylate pH 7 and 120 mM KCl, or 100 µM RNA strand concentration (NMR) dissolved in 20 mM Potassium phosphate pH7 and 120 mM KCl: **a)** Thermal melting transition measured at 295 nm . b) Thermal melting profiles measured at 260 nm **. c) Thermal** differential Spectra (TDS). **d)** Circular Dichroism (CD) spectra. **e)** 1D 1H-NMR spectrum of the imino proton region recorded at 15°C.

Figure S16A

Name	Sequence (5'-3')[a]	Length (nt)	RNA Strand +/-	Position (nt)	CD[b]	TDS[c] +/-	T_m (°C)[d]	NMR (YES/NO)	G4?[f] YES/NO
S1-6	UGGAGGACGCAAUGGGGCAAGGC	23	(+)	28204	folded	(+)	35/40 (295 nm)<br>55 (260 nm)	nd	yes (g4<->hp)

a. Sequence retrieved from the reference isolate for each virus

b. CD: circular dichroism. Folded/unfolded depending on the intensity of the CD spectra. nd: not determined

c.TDS: Thermal differential spectrum. (+) indicates the presence of a peak at 295 nm. (-) indicate the absence of the peak.

d.T_m: Thermal melting temperature (the standard error is of ±1°C). Two Tm values are indicated when the melting process presents an hysteresis.

e. NMR: Yes indicates the presence of imino proton resonances. No indicates the absence of imino protons resonances

f. G4? Yes or No indicate that the sequence forms or not a G4 according to the data of CD, TDS, Tm. Hp means that a RNA hairpin structure is predicted by VIENNA software. G4<->hp : we speculate a G4/Hairpin equilibrium

Figure S16B: Biophysical *In vitro* characterization

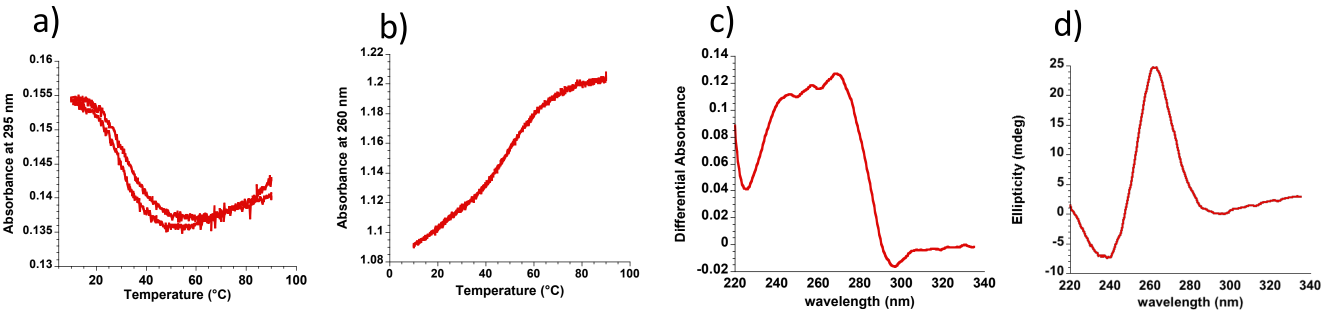

e)

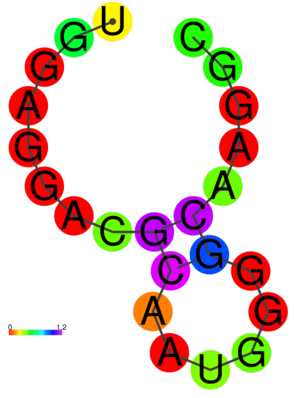

Analysis: T_m: At 295 nm, the hypochromic melting process present an hysteresis of 5°C. At 260 nm, the melting temperature is higher and the process is reversible suggesting that another mono-molecular structure is formed. **TDS** : The hypochromicity at 295 nm suggests the formation of a G4 fold. **CD** : The intense positive peak at 267 nm suggest that the RNA oligonucleotide is well folded. **Prediction:** hairpin like structure.

Experiments were performed at around 4 µM (CD, TDS, UV-melting) dissolved in 10 mM lithium cacodylate pH 7 and 120 mM KCl : **a)** Thermal melting transition measured at 295 nm . b) Thermal melting profiles measured at 260 nm **. c)** Thermal differential Spectra (TDS). **d)** Circular Dichroism (CD) spectra **. e)** Structure prediction using the RNA fold prediction on the vienna RNA websuite.

Figure S17A

Name	Sequence (5'-3')[a]	Length (nt)	RNA Strand +/-	Position (nt)	CD[b]	TDS[c] +/-	T_m (°C)[d]	NMR (YES/NO)	G4?[f] YES/NO
S1-7	UGGGUAAACCUUGGGGUCGGCGCUGUUUUGGC	32	(-)	28229	folded	(-)	47 (260 nm)	nd	no (hp)

a. Sequence retrieved from the reference isolate for each virus
b. CD: circular dichroism. Folded/unfolded depending on the intensity of the CD spectra. nd: not determined
c.TDS: Thermal differential spectrum. (+) indicates the presence of a peak at 295 nm. (-) indicate the absence of the peak.
d.T_m: Thermal melting temperature (the standard error is of ±1°C). Two Tm values are indicated when the melting process presents an hysteresis.
e. NMR: Yes indicates the presence of imino proton resonances. No indicates the absence of imino protons resonances
f. G4? Yes or No indicate that the sequence forms or not a G4 according to the data of CD, TDS, Tm. Hp means that a RNA hairpin structure is predicted by VIENNA software. G4<->hp : we speculate a G4/Hairpin equilibrium

Figure S17B: Biophysical *In vitro* characterization

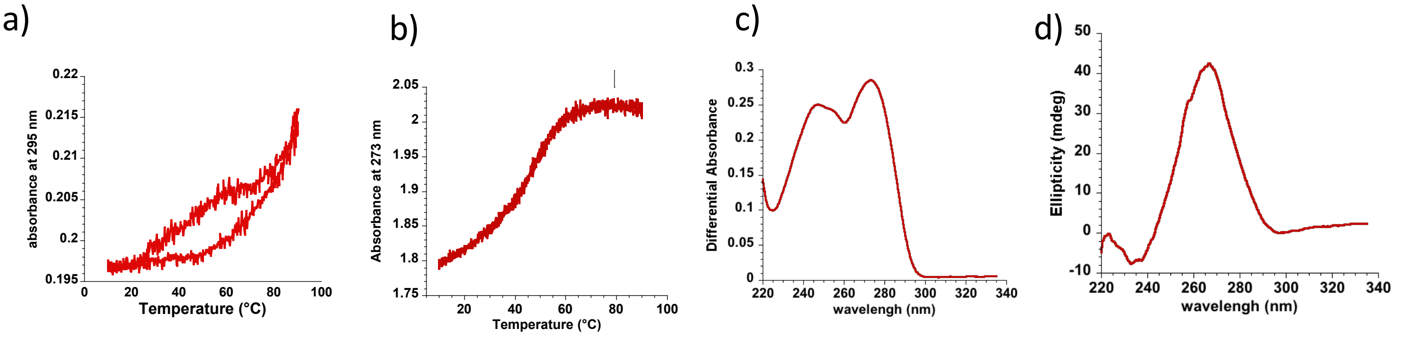

e)

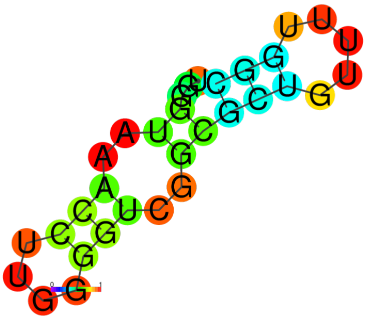

Analysis: T_m: The melting profile at 295 nm does not present any hypochromism nor a structural transition. At 260 nm, the melting presents a clear sigmoidal structural transition. The absence of hysteresis suggest that the structures is mono-molecular. **TDS** : The absence of hypochromicity at 295 nm argue for a non-G4 structure. **CD** : The intense positive peak at 268 nm suggest that the structure is folded. **Prediction:** Hairpin like structure

Experiments were performed at around 4 µM (CD, TDS, UV-melting) dissolved in 10 mM lithium cacodylate pH 7 and 120 mM KCl l: **a)** Thermal melting transition measured at 295 nm . **b)** Thermal melting profiles measured at 260 nm **. c) Thermal** differential Spectra (TDS). **d)** Circular Dichroism (CD) spectra **. e)** Structure prediction using the RNA fold prediction on the vienna RNA websuite.

Figure S18A

Name	Sequence (5'-3')[a]	Length (nt)	RNA Strand +/-	Position (nt)	CD[b]	TDS[c] +/-	T_m (°C)[d]	NMR (YES/NO)	G4?[f] YES/NO
M-1	GGGUUUGCCUGUGGAUGUGGGG	22	(+)	1337	folded	(+)	42/56 (295 nm) 50/53 (260 nm)	nd	yes (g4<->hp)

a. Sequence retrieved from the reference isolate for each virus
b. CD: circular dichroism. Folded/unfolded depending on the intensity of the CD spectra. nd: not determined
c.TDS: Thermal differential spectrum. (+) indicates the presence of a peak at 295 nm. (-) indicate the absence of the peak.
d.T_m: Thermal melting temperature (the standard error is of ±1°C). Two Tm values are indicated when the melting process presents an hysteresis.
e. NMR: Yes indicates the presence of imino proton resonances. No indicates the absence of imino protons resonances
f. G4? Yes or No indicate that the sequence forms or not a G4 according to the data of CD, TDS, Tm. Hp means that a RNA hairpin structure is predicted by VIENNA software. G4<->hp : we speculate a G4/Hairpin equilibrium

Figure S18B: Biophysical *In vitro* characterization

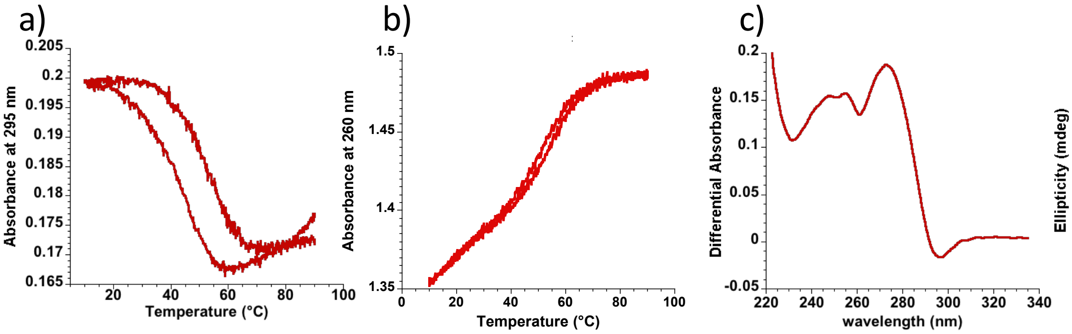

e)

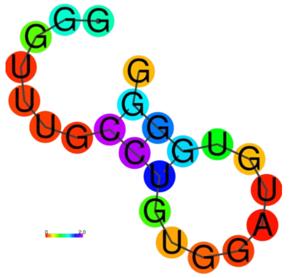

Analysis: T_m : At 295 nm, the process is not reversible. The heating and cooling profiles have 2 different melting temperatures. This hysteresis of 14°C shows that the folding and unfolding processes are kinetically slow suggesting the presence of a majority of multi-molecular structures. At 260 nm, the process presents a cooperative structural transition with a little hysteresis of 3°C suggesting a mono-molecular secondary structure. An equilibrium between a bimolecular G4 and an mono-molecular hairpin is possible. **TDS**: The hypochromicity around 295 nm suggests the formation of a G4 fold. **CD** : The intense positive peaks at 267 nm suggests the formation of a stable structure. **Prediction:** Hairpin like structure

Experiments were performed at around 4 µM (CD, TDS, UV-melting) dissolved in 10 mM lithium cacodylate pH 7 and 120 mM KCl : **a)** Thermal melting transition measured at 295 nm . **b)** Thermal melting profiles measured at 260 nm **. c)** Thermal differential Spectra (TDS). **d)** Circular Dichroism (CD) spectra **. e)** Structure prediction using the RNA fold prediction on the vienna RNA websuite.

Figure S19A

Name	Sequence (5'-3')[a]	Length (nt)	RNA Strand +/-	Position (nt)	CD[b]	TDS[c] +/-	T_m (°C)[d]	NMR (YES/NO)	G4?[f] YES/NO
M-2	GGGUUUGUGGUGGUCAAUGG	20	(+)	2345	folded	(+)	46/56 (295 nm)	YES	YES

a. Sequence retrieved from the reference isolate for each virus
b. CD: circular dichroism. Folded/unfolded depending on the intensity of the CD spectra. nd: not determined
c.TDS: Thermal differential spectrum. (+) indicates the presence of a peak at 295 nm. (-) indicate the absence of the peak.
d.T_m: Thermal melting temperature (the standard error is of ±1°C). Two Tm values are indicated when the melting process presents an hysteresis.
e. NMR: Yes indicates the presence of imino proton resonances. No indicates the absence of imino protons resonances
f. G4? Yes or No indicate that the sequence forms or not a G4 according to the data of CD, TDS, Tm. Hp means that a RNA hairpin structure is predicted by VIENNA software. G4<->hp : we speculate a G4/Hairpin equilibrium

Figure S19B: Biophysical *In vitro* characterization

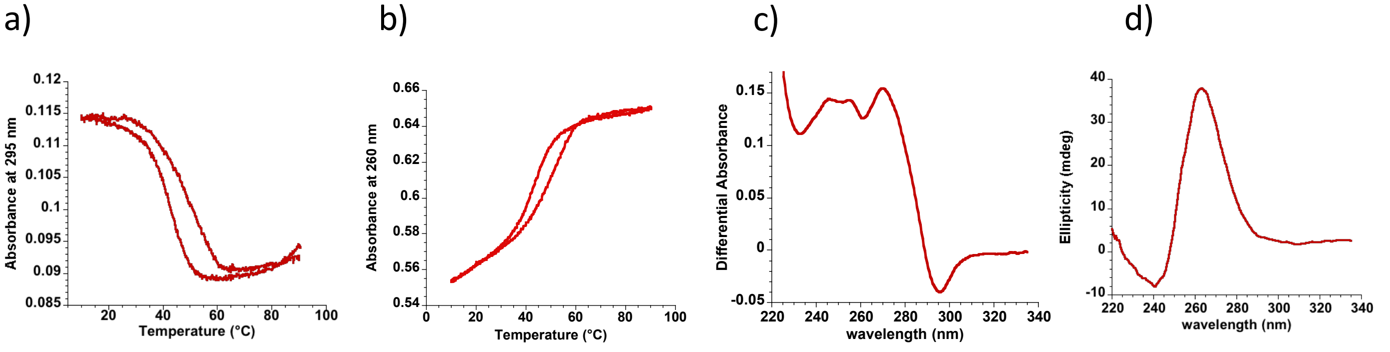

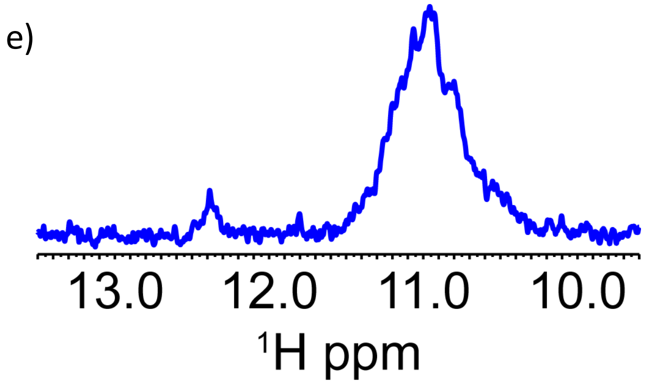

Analysis: T_m: at both 295 nm and 260 nm, the process is not reversible and presents an hysteresis of 10°C. The structure is multi-molecular. **TDS** : The hypochromicity at 295 nm suggests the formation of a G4 fold. **CD** : The intense positive peak at 267 nm suggest that the structure is folded. **NMR:** Presence of a very broad NMR signal in the [10 – 12] ppm suggesting the formation of highly polymorphic G4 structures. Peaks in the [12 – 13] ppm region suggests the formation of additional CG base pairs

Experiments were performed at around 4 µM (CD, TDS, UV-melting) dissolved in 10 mM lithium cacodylate pH 7 and 120 mM KCl, or 100 µM RNA strand concentration (NMR) dissolved in 20 mM Potassium phosphate pH7 and 120 mM KCl: **a)** Thermal melting transition measured at 295 nm . b) Thermal melting profiles measured at 260 nm **. c)** Thermal differential Spectra (TDS). **d)** Circular Dichroism (CD) spectra. **e)** 1D 1H-NMR spectrum of the imino proton region recorded at 15°C.

Figure S20A

Name	Sequence (5'-3')[a]	Length (nt)	RNA Strand +/-	Position (nt)	CD[b]	TDS[c] +/-	T_m (°C)[d]	NMR (YES/NO)	G4?[f] YES/NO
M-3	GUGGAAGAUGGUUGUGUGUG	22	(+)	5020	FOLDED	(+)	38 (295 nm)	YES	YES

a. Sequence retrieved from the reference isolate for each virus
b. CD: circular dichroism. Folded/unfolded depending on the intensity of the CD spectra. nd: not determined
c.TDS: Thermal differential spectrum. (+) indicates the presence of a peak at 295 nm. (-) indicate the absence of the peak.
d.T_m: Thermal melting temperature (the standard error is of ±1°C). Two Tm values are indicated when the melting process presents an hysteresis.
e. NMR: Yes indicates the presence of imino proton resonances. No indicates the absence of imino protons resonances
f. G4? Yes or No indicate that the sequence forms or not a G4 according to the data of CD, TDS, Tm. Hp means that a RNA hairpin structure is predicted by VIENNA software. G4<->hp : we speculate a G4/Hairpin equilibrium

Figure S20B: Biophysical *In vitro* characterization

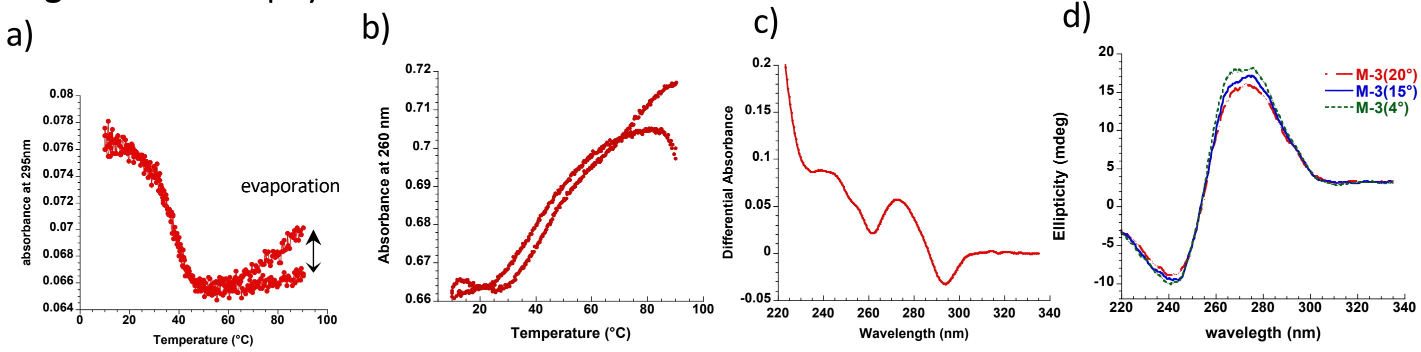

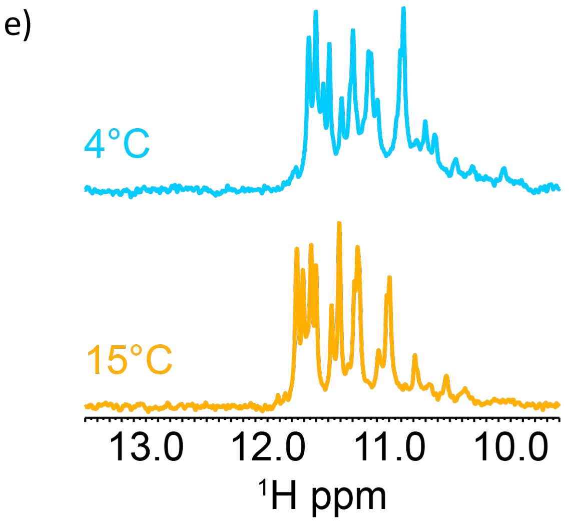

Analysis: T_m: At 295 nm, the process is reversible, no hysteresis is detected. The heating and cooling profiles have the same melting temperatures. The structure is mono-molecular. At 260 nm, the melting profile presents an hysteresis of 10°C. **TDS** : A slight hypochromicity at 295 nm suggests the formation of a G4 fold. **CD** : The positive peak at 267 nm suggest that the oligonucleotide is folded. NMR: the presence of a set of well resolved peak in the [10 – 12] ppm region confirms the formation of G4s with a limited conformational polymorphism at both 4°C and 15°C.

Experiments were performed at around 4 µM (CD, TDS, UV-melting) dissolved in 10 mM lithium cacodylate pH 7 and 120 mM KCl, or 100 µM RNA strand concentration (NMR) dissolved in 20 mM Potassium phosphate pH7 and 120 mM KCl: **a)** Thermal melting transition measured at 295 nm . **b)** Thermal melting profiles measured at 260 nm **. c) Thermal** differential Spectra (TDS). **d)** Circular Dichroism (CD) spectra recorded at 20°C, 15°C and 4°C. **e)** 1D 1H-NMR spectrum of the imino proton region recorded at 15°C and 4°C.

Figure S21A

Name	Sequence (5'-3')[a]	Length (nt)	RNA Strand +/-	Position (nt)	CD[b]	TDS[c] +/-	T_m (°C)[d]	NMR (YES/NO)	G4?[f] YES/NO
M-4	AGGGGGCUGCGUGGC	15	(+)	18461	FOLDED	(-)	47/52 (260 nm)	ND	NO (HP)

a. Sequence retrieved from the reference isolate for each virus
b. CD: circular dichroism. Folded/unfolded depending on the intensity of the CD spectra. nd: not determined
c.TDS: Thermal differential spectrum. (+) indicates the presence of a peak at 295 nm. (-) indicate the absence of the peak.
d.T_m: Thermal melting temperature (the standard error is of ±1°C). Two Tm values are indicated when the melting process presents an hysteresis.
e. NMR: Yes indicates the presence of imino proton resonances. No indicates the absence of imino protons resonances
f. G4? Yes or No indicate that the sequence forms or not a G4 according to the data of CD, TDS, Tm. Hp means that a RNA hairpin structure is predicted by VIENNA software. G4<->hp : we speculate a G4/Hairpin equilibrium

Figure S21B: Biophysical *In vitro* characterization

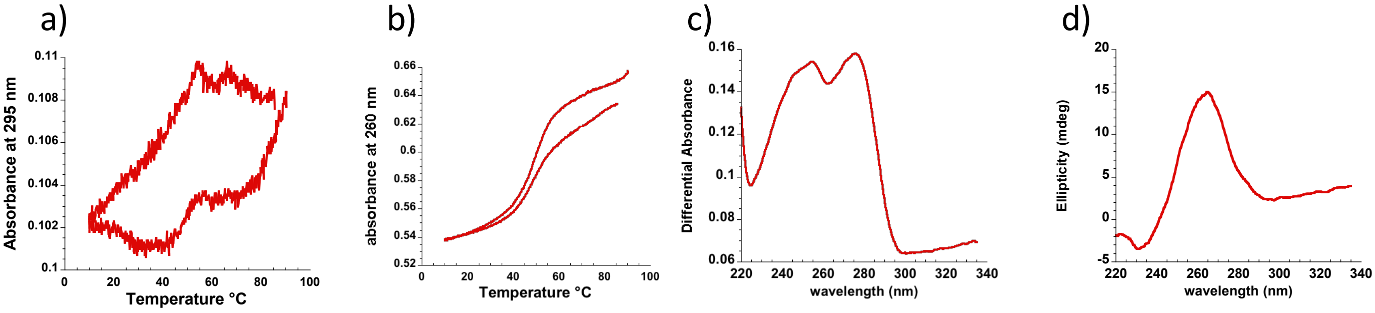

e)

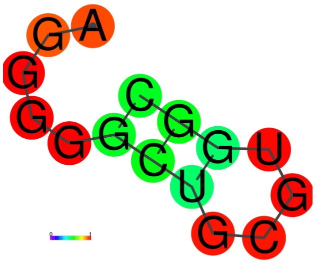

Analysis: T_m: The melting profile at 295 nm does not present any hypochromism nor a structural transition. At 260 nm, the melting profile presents a clear sigmoidal structural transition. **TDS** : The absence of hypochromicity at 295 nm argue for a non-G4 fold. **CD** : The intense positive peak at 268 nm suggests that the structure is folded. **Prediction:** Hairpin like structure

Experiments were performed at around 4 µM (CD, TDS, UV-melting) dissolved in 10 mM lithium cacodylate pH 7 and 120 mM KCl : **a)** Thermal melting transition measured at 295 nm . **b)** Thermal melting profiles measured at 260 nm **. c)** Thermal differential Spectra (TDS). **d)** Circular Dichroism (CD) spectra **. e)** Structure prediction using the RNA fold prediction on the vienna RNA websuite.

Figure S22A

Name	Sequence (5’-3’)[a]	Length (nt)	RNA Strand +/-	Position (nt)	CD[b]	TDS[c] +/-	T_m (°C)[d]	NMR (YES/NO)	G4?[f] YES/NO
M-5	AGAGGAGGAGGGAGGU	16	(-)	25104	folded	(+)	63 (295 nm)	yes	yes

a. Sequence retrieved from the reference isolate for each virus
b. CD: circular dichroism. Folded/unfolded depending on the intensity of the CD spectra. nd: not determined
c.TDS: Thermal differential spectrum. (+) indicates the presence of a peak at 295 nm. (-) indicate the absence of the peak.
d.T_m: Thermal melting temperature (the standard error is of ±1°C). Two Tm values are indicated when the melting process presents an hysteresis.
e. NMR: Yes indicates the presence of imino proton resonances. No indicates the absence of imino protons resonances
f. G4? Yes or No indicate that the sequence forms or not a G4 according to the data of CD, TDS, Tm. Hp means that a RNA hairpin structure is predicted by VIENNA software. G4<->hp : we speculate a G4/Hairpin equilibrium

Figure S22B: Biophysical *In vitro* characterization

a)

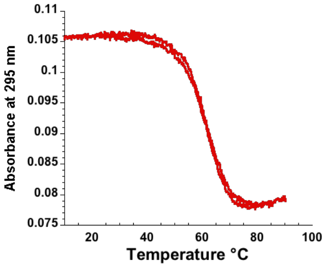

b)

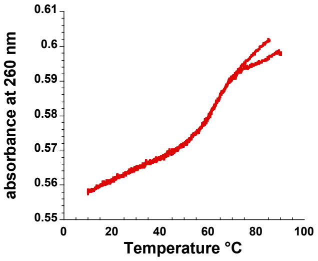

c)

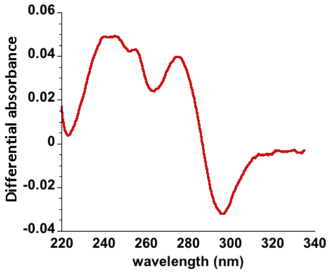

d)

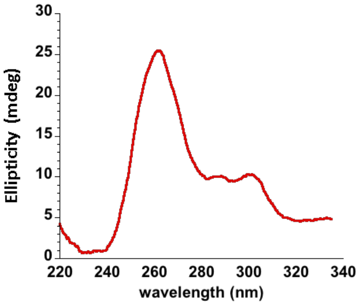

e)

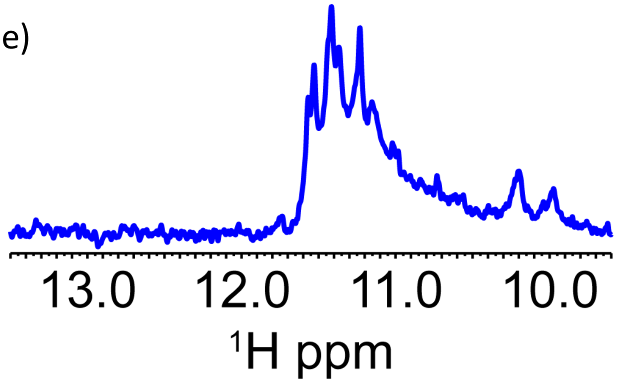

Analysis: T_m: At 295 nm, the structure is stable and the melting process presents a cooperative and reversible transition. At 260 nm, the process is also reversible and presents a cooperative sigmoidal transition. **TDS** : The hypochromicity at 295 nm suggests the formation of a G4 fold. **CD** : The intense positive peak at 267 nm suggest that the oligo is folded. NMR: the presence of a set of well resolved peak in the [10 – 12] ppm region confirms the formation of G4s with a limited conformational polymorphism.

Experiments were performed at around 4 µM (CD, TDS, UV-melting) dissolved in 10 mM lithium cacodylate pH 7 and 120 mM KCl, or 100 µM RNA strand concentration (NMR) dissolved in 20 mM Potassium phosphate pH7 and 120 mM KCl: **a)** Thermal melting transition measured at 295 nm . **b)** Thermal melting profiles measured at 260 nm **. c) Thermal** differential Spectra (TDS). **d)** Circular Dichroism (CD) spectra. **e)** 1D 1H-NMR spectrum of the imino proton region recorded at 15°C.

Figure S23A

Name	Sequence (5'-3')[a]	Length (nt)	RNA Strand +/-	Position (nt)	CD[b]	TDS[c] +/-	T_m (°C)[d]	NMR (YES/NO)	G4?[f] YES/NO
M-6	UGGGACUAGCUGGACGGGA	19	(-)	26865	folded	(-)	40 (260 nm)	nd	no

a. Sequence retrieved from the reference isolate for each virus
b. CD: circular dichroism. Folded/unfolded depending on the intensity of the CD spectra. nd: not determined
c.TDS: Thermal differential spectrum. (+) indicates the presence of a peak at 295 nm. (-) indicate the absence of the peak.
d.T_m: Thermal melting temperature (the standard error is of ±1°C). Two Tm values are indicated when the melting process presents an hysteresis.
e. NMR: Yes indicates the presence of imino proton resonances. No indicates the absence of imino protons resonances
f. G4? Yes or No indicate that the sequence forms or not a G4 according to the data of CD, TDS, Tm. Hp means that a RNA hairpin structure is predicted by VIENNA software. G4<->hp : we speculate a G4/Hairpin equilibrium

Figure S23B: Biophysical *In vitro* characterization

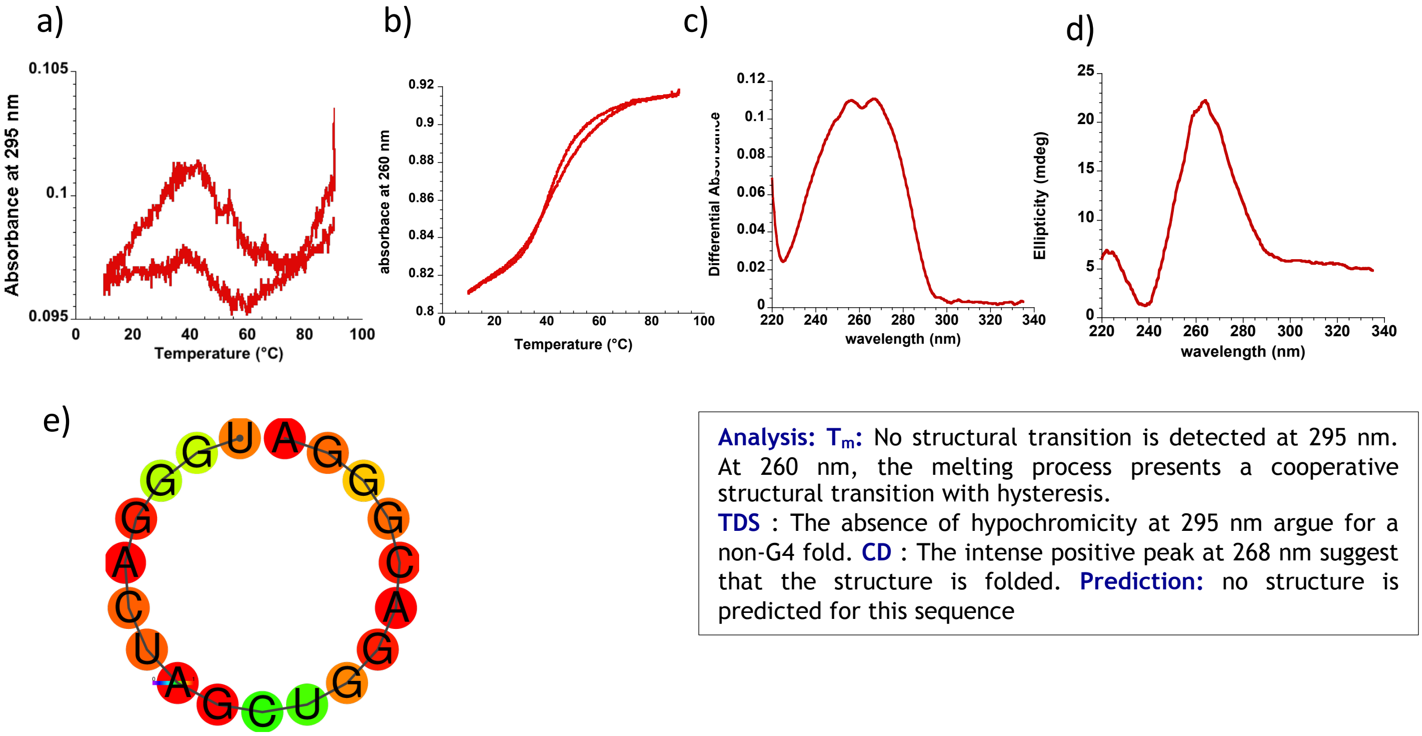

Analysis: T_m: No structural transition is detected at 295 nm. At 260 nm, the melting process presents a cooperative structural transition with hysteresis.
TDS : The absence of hypochromicity at 295 nm argue for a non-G4 fold. **CD** : The intense positive peak at 268 nm suggest that the structure is folded. **Prediction:** no structure is predicted for this sequence

Experiments were performed at around 4 µM (CD, TDS, UV-melting) dissolved in 10 mM lithium cacodylate pH 7 and 120 mM KCl : **a)** Thermal melting transition measured at 295 nm . **b)** Thermal melting profiles measured at 260 nm **. c) Thermal** differential Spectra (TDS). **d)** Circular Dichroism (CD) spectra. **e)** Structure prediction using the RNA fold prediction on the vienna RNA websuite.

Figure S24A

Name	Sequence (5'-3')[a]	Length (nt)	RNA Strand +/-	Position (nt)	CD[b]	TDS[c] +/-	T_m (°C)[d]	NMR (YES/NO)	G4?[f] YES/NO
M-7	GGGUUUGUGGUGGUCAAUGG	20	(-)	27807	folded	(+)	61/79 (295 nm)	yes	yes

a. Sequence retrieved from the reference isolate for each virus
b. CD: circular dichroism. Folded/unfolded depending on the intensity of the CD spectra. nd: not determined
c.TDS: Thermal differential spectrum. (+) indicates the presence of a peak at 295 nm. (-) indicate the absence of the peak.
d.T_m: Thermal melting temperature (the standard error is of ±1°C). Two Tm values are indicated when the melting process presents an hysteresis.
e. NMR: Yes indicates the presence of imino proton resonances. No indicates the absence of imino protons resonances
f. G4? Yes or No indicate that the sequence forms or not a G4 according to the data of CD, TDS, Tm. Hp means that a RNA hairpin structure is predicted by VIENNA software. G4<->hp : we speculate a G4/Hairpin equilibrium

Figure S24B: Biophysical *In vitro* characterization

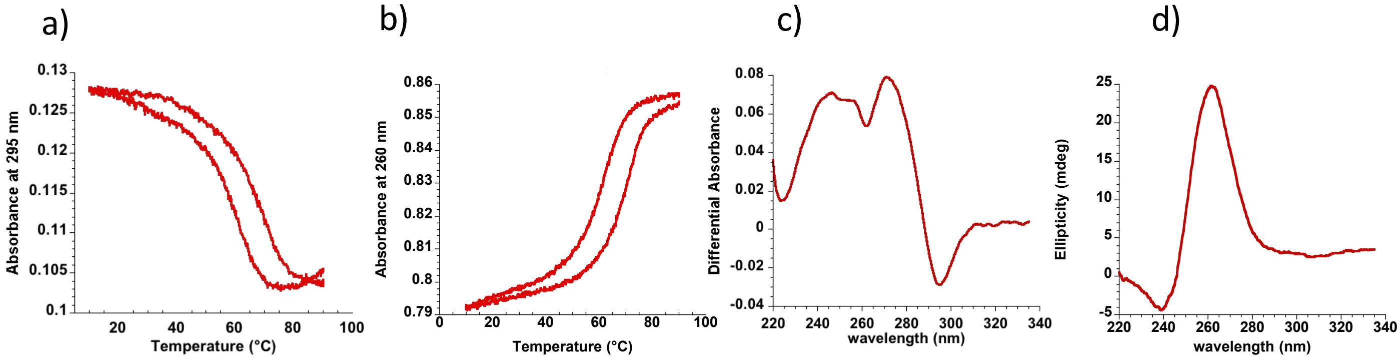

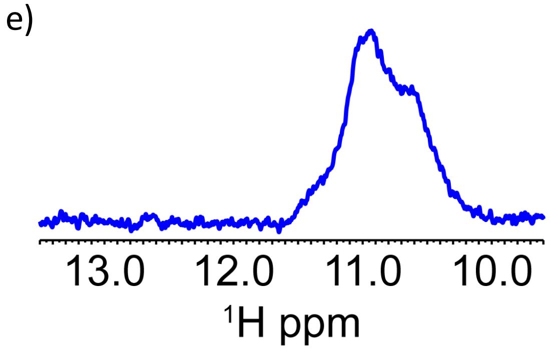

Analysis: T_m: At both 295 nm and 260 nm, the process is not reversible and presents an hysteresis of 18°C. The structure is multi-molecular. **TDS** : The hypochromicity at 295 nm suggests the formation of a G4 fold. **CD** : The intense positive peak at 267 nm suggest that the structure is folded. **NMR:** Presence of a very broad NMR signal in the [10 – 12] ppm suggesting the formation of highly polymorphic G4 structures.

Experiments were performed at around 4 µM (CD, TDS, UV-melting) dissolved in 10 mM lithium cacodylate pH 7 and 120 mM KCl, or 100 µM RNA strand concentration (NMR) dissolved in 20 mM Potassium phosphate pH7 and 120 mM KCl: **a)** Thermal melting transition measured at 295 nm . b) Thermal melting profiles measured at 260 nm **. c) Thermal** differential Spectra (TDS). **d)** Circular Dichroism (CD) spectra. **e)** 1D 1H-NMR spectrum of the imino proton region recorded at 15°C.

Figure S25A

Name	Sequence (5'-3')[a]	Length (nt)	RNA Strand +/-	Position (nt)	CD[b]	TDS[c] +/-	T_m (°C)[d]	NMR (YES/NO)	G4?[f] YES/NO
M-8	AGGUGGAAAGGUAAGAGGGAG	21	(-)	28721	folded	(+)	36/44 (295 nm)	yes	yes

a. Sequence retrieved from the reference isolate for each virus

b. CD: circular dichroism. Folded/unfolded depending on the intensity of the CD spectra. nd: not determined

c.TDS: Thermal differential spectrum. (+) indicates the presence of a peak at 295 nm. (-) indicate the absence of the peak.

d.T_m: Thermal melting temperature (the standard error is of ±1°C). Two Tm values are indicated when the melting process presents an hysteresis.

e. NMR: Yes indicates the presence of imino proton resonances. No indicates the absence of imino protons resonances

f. G4? Yes or No indicate that the sequence forms or not a G4 according to the data of CD, TDS, Tm. Hp means that a RNA hairpin structure is predicted by VIENNA software. G4<->hp : we speculate a G4/Hairpin equilibrium

Figure S25B: Biophysical *In vitro* characterization

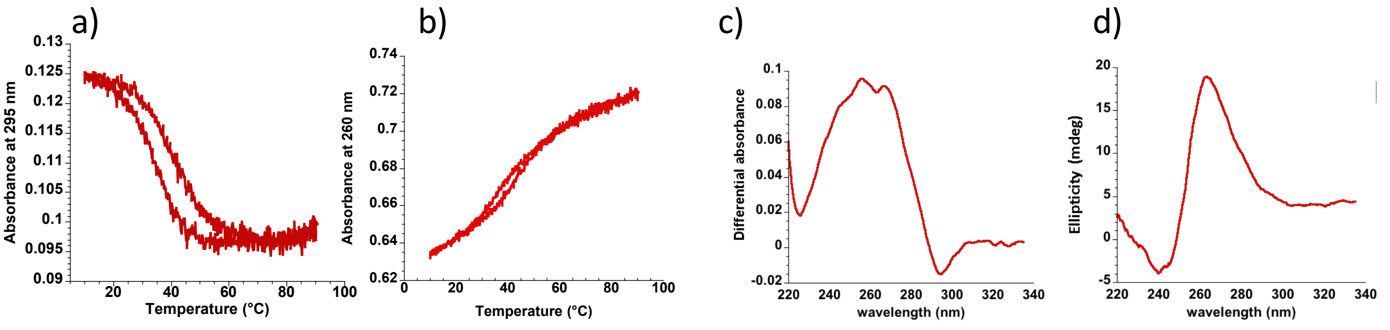

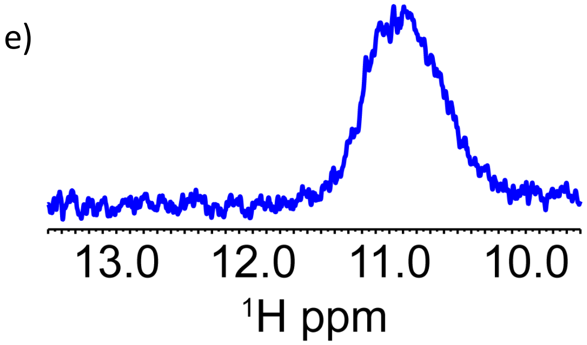

Analysis: T_m: at both 295 nm and 260 nm, the process is not reversible and presents an hysteresis of 8°C. The structure is multi-molecular. **TDS** : The hypochromicity at 295 nm suggests the formation of a G4 fold. **CD** : The intense positive peak at 267 nm suggest that the structure is folded. **NMR:** Presence of a very broad NMR signal in the [10 – 12] ppm suggesting the formation of highly polymorphic G4 structures.

Experiments were performed at around 4 µM (CD, TDS, UV-melting) dissolved in 10 mM lithium cacodylate pH 7 and 120 mM KCl, or 100 µM RNA strand concentration (NMR) dissolved in 20 mM Potassium phosphate pH7 and 120 mM KCl: **a)** Thermal melting transition measured at 295 nm . b) Thermal melting profiles measured at 260 nm **. c) Thermal** differential Spectra (TDS). **d)** Circular Dichroism (CD) spectra. **e)** 1D 1H-NMR spectrum of the imino proton region recorded at 15°C.

Figure S26A

Name	Sequence (5'-3')[a]	Length (nt)	RNA Strand +/-	Position (nt)	CD[b]	TDS[c] +/-	T_m (°C)[d]	NMR (YES/NO)	G4?[f] YES/NO
M-9	UGGGUAUUGGUGGAGACAGGA	21	(+)	28789	folded	(-)	25 (260 nm)	nd	no

a. Sequence retrieved from the reference isolate for each virus

b. CD: circular dichroism. Folded/unfolded depending on the intensity of the CD spectra. nd: not determined

c.TDS: Thermal differential spectrum. (+) indicates the presence of a peak at 295 nm. (-) indicate the absence of the peak.

d.T_m: Thermal melting temperature (the standard error is of ±1°C). Two Tm values are indicated when the melting process presents an hysteresis.

e. NMR: Yes indicates the presence of imino proton resonances. No indicates the absence of imino protons resonances

f. G4? Yes or No indicate that the sequence forms or not a G4 according to the data of CD, TDS, Tm. Hp means that a RNA hairpin structure is predicted by VIENNA software. G4<->hp : we speculate a G4/Hairpin equilibrium

Figure S26B: Biophysical *In vitro* characterization

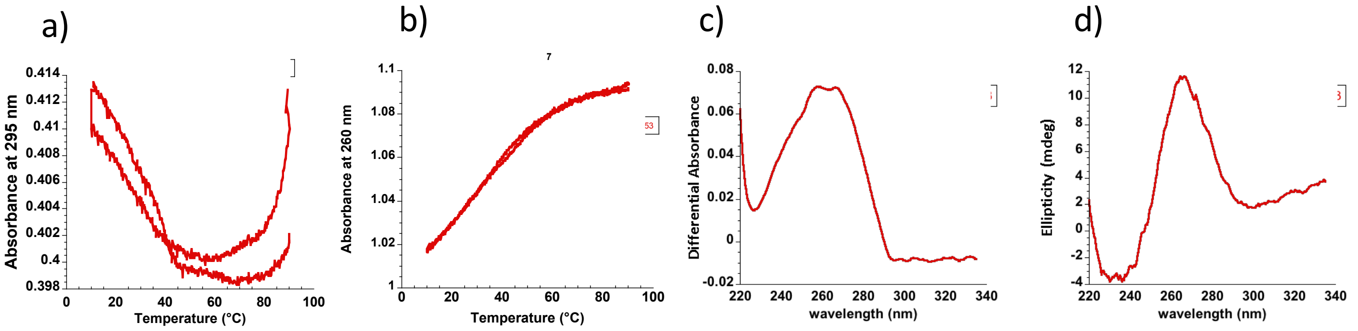

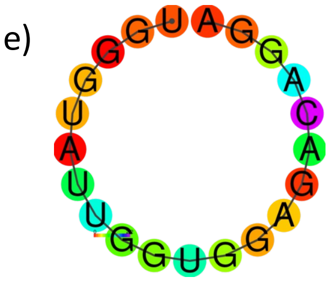

Analysis: T_m: No structural transition is detected at 295 nm. At 260 nm, the melting process presents a very poor structural transition. **TDS** : The absence of hypochromicity at 295 nm argue for a non-G4 fold. **CD** : The weak CD peak suggest that the structure is mostly unfolded at 20°C. **Prediction:** no structure can be predicted for this sequence

Experiments were performed at around 4 µM (CD, TDS, UV-melting) dissolved in 10 mM lithium cacodylate pH 7 and 120 mM KCl l: **a)** Thermal melting transition measured at 295 nm . **b)** Thermal melting profiles measured at 260 nm . **c)** Thermal differential Spectra (TDS). **d)** Circular Dichroism (CD) spectra. **e)** Structure prediction using the RNA fold prediction on the vienna RNA websuite.

Figure S27A

Name	Sequence (5'-3')[a]	Length (nt)	RNA Strand +/-	Position (nt)	CD[b]	TDS[c] +/-	T_m (°C)[d]	NMR (YES/NO)	G4?[f] YES/NO
M-10	AGGGGACUGGAGGC	14	(+)	29045	folded	(+)	41/59 (295 nm)	yes	yes

a. Sequence retrieved from the reference isolate for each virus

b. CD: circular dichroism. Folded/unfolded depending on the intensity of the CD spectra. nd: not determined

c.TDS: Thermal differential spectrum. (+) indicates the presence of a peak at 295 nm. (-) indicate the absence of the peak.

d.T_m: Thermal melting temperature (the standard error is of ±1°C). Two Tm values are indicated when the melting process presents an hysteresis.

e. NMR: Yes indicates the presence of imino proton resonances. No indicates the absence of imino protons resonances

f. G4? Yes or No indicate that the sequence forms or not a G4 according to the data of CD, TDS, Tm. Hp means that a RNA hairpin structure is predicted by VIENNA software. G4<->hp : we speculate a G4/Hairpin equilibrium

Figure S27B: Biophysical *In vitro* characterization

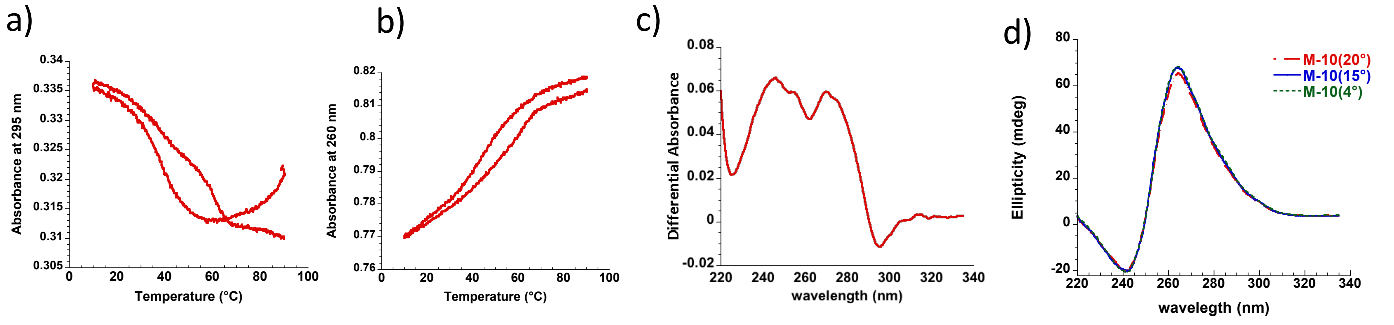

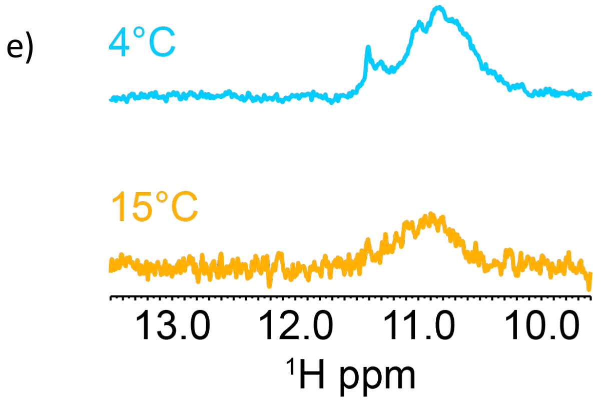

Analysis: T_m: at both 295 nm and 260 nm, the process is not reversible and presents an hysteresis of 10°C. The structure is multi-molecular. **TDS** : The hypochromicity at 295 nm suggests the formation of a G4 fold. **CD** : The intense positive peak at 267 nm suggest that the structure is folded. **NMR:** At 4°C, imino proton peaks were detected in the [10 – 12] ppm region. At 15°C, this G4 start to melt confirming the low stability measured by UV-melting.

Experiments were performed at around 4 µM (CD, TDS, UV-melting) dissolved in 10 mM lithium cacodylate pH 7 and 120 mM KCl, or 100 µM RNA strand concentration (NMR) dissolved in 20 mM Potassium phosphate pH7 and 120 mM KCl: **a)** Thermal melting transition measured at 295 nm . b) Thermal melting profiles measured at 260 nm **. c)** Thermal differential Spectra (TDS). d) Circular Dichroism (CD) spectra recorded at 20°C, 15°C and 4°C. **e)** 1D 1H-NMR spectrum of the imino proton region recorded at 15°C and 4°C.
